# Species diversity of Pleosporalean taxa associated with *Camellia sinensis* (L.) Kuntze in Taiwan

**DOI:** 10.1038/s41598-020-69718-0

**Published:** 2020-07-29

**Authors:** Hiran A. Ariyawansa, Ichen Tsai, Kasun M. Thambugala, Wei-Yu Chuang, Shiou-Ruei Lin, Wael N. Hozzein, Ratchadawan Cheewangkoon

**Affiliations:** 1 Department of Plant Pathology and Microbiology, College of Bio-Resources and Agriculture, National Taiwan University, Taipei City, 10617 Taiwan; 2 Genetics and Molecular Biology Unit, Faculty of Applied Sciences, University of Sri Jayewardenepura, Gangodawila, Nugegoda, Sri Lanka; 3 Department of Tea Agronomy, Tea Research and Extension Station, Taoyuan City, 32654 Taiwan; 4 Bioproducts Research Chair, Zoology Department, College of Science, King Saud University, Riyadh, 11451 Saudi Arabia; 5 Botany and Microbiology Department, Faculty of Science, Beni-Suef University, Beni-Suef, 62521 Egypt; 6 Department of Entomology and Plant Pathology, Faculty of Agriculture, Chiang Mai University, Chiang Mai, 50200 Thailand; 7 Innovative Agriculture Research Centre, Faculty of Agriculture, Chiang Mai University, Chiang Mai, 50200 Thailand

**Keywords:** Biotechnology, Microbiology

## Abstract

Pleosporales species are important plant pathogens, saprobes, and endophytes on a wide range of economically important plant hosts. The classification of Pleosporales has undergone various modifications in recent years due to the addition of many families described from multiple habitats with a high level of morphological deviation. Numerous asexual genera have been described in Pleosporales that can be either hyphomyceteous or coelomycetous. Phoma- or coniothyrium-like species are common and have been revealed as polyphyletic in the order Pleosporales and linked with several sexual genera. A total of 31 pleosporalean strains were isolated in different regions of Taiwan between 2017 and 2018 from the leaves of *Camellia sinensis* plants with symptoms of leaf spot disease. These strains were evaluated morphologically and genotypically using multi-locus sequence analyses of the ITS, LSU, SSU, *rpb*2, *tef*1 and *tub*2 genes. The results demonstrated the affiliation of these strains with the various families in Pleosporales and revealed the presence of one new genus (*Neoshiraia*) and eight new species (*Alloconiothyrium camelliae*, *Amorocoelophoma camelliae*, *Leucaenicola camelliae*, *L*. *taiwanensis*, *Neoshiraia camelliae*, *N*. *taiwanensis*, *Paraconiothyrium camelliae* and *Paraphaeosphaeria camelliae*). Furthermore, to the best of our understanding, *Didymella segeticola*, *Ectophoma pomi* and *Roussoella mexican* were reported for the first time from *C. sinensis* in Taiwan.

## Introduction

Pleosporales is the largest and most diverse order of the class Dothideomycetes, comprising over 4,700 species recognised in more than 50 families^1–5^. Pleosporalean taxa are characterized by pseudothecial ascomata typically with a papilla, and an ascomatal wall composed of several layers of cells^4,6–9^. Asci are bitunicate, regularly fissitunicate and born within a persistent hamathecium with or without pseudoparaphyses bearing mostly septate ascospores but that vary in colour and shape, with or without a gelatinous sheath^4,6–9^. Various asexual morphs are produced in Pleosporales that can be either coelomycetous or hyphomycetous^3–5,8–10^. The order Pleosporales contains saprobic, endophytic and pathogenic species allied with an extensive range of hosts and substrates^1,2,9^.

In recent years, molecular studies have revealed multiple non-monophyletic genera within Pleosporales, and clades of pleosporalean species that do not always correlate to species groups based on morphological characteristics. Recently, many new pleosporalean lineages from freshwater^8^ or marine^9^ environments or from bambusicolous hosts^9^ have been reported. Pleosporales contains two major suborders, Pleosporineae and Massarineae^4,8,9^. Massarineae was introduced by Zhang et al.^8^ and currently comprises 12 families^11^. The suborder Pleosporineae comprises several economically significant plant and human pathogens, and at present, comprises 21 families^1,2,5,11^.

Tea is the most widely consumed liquid in the world after water. *Camellia sinensis* (L.) Kuntze plants, widely grown in the tropics and subtropics, are commonly processed to produce tea^12^. To meet increasing demand, tea cultivation has been expanded in many countries in tropical and subtropical regions including Taiwan. According to the USDA database, 520 fungal taxa have been identified as associated with *Camellia* spp., of which 303 were from *C. sinensis*^12^. Various pleosporalean taxa have been reported from *Camellia* species. i.e.* Alternaria camelliae* (Cooke & Massee) P. Joly., *Paraboeremia camelliae* J.R. Jiang & L. Cai., *Pleospora camelliae* Dippen., and *Phoma camelliae* Cooke^3–5,8–10^.

Multiple studies have been conducted to elucidate the diversity of pleosporalean fungi associated with various hosts and habitats in Taiwan^1,2,13–17^ but up to now, no inclusive study has been carried out on pleosporalean linages allied with *C. sinensis*. The main aim of the present survey is to fill this gap in the information of populations of pleosporalean taxa on *C. sinensis* in the major tea production provinces in Taiwan and confirm their natural classification via morphology coupled with a phylogenetic analysis of single- and multi-locus sequence data.

## Results

### Phylogeny

**Figure 1:**
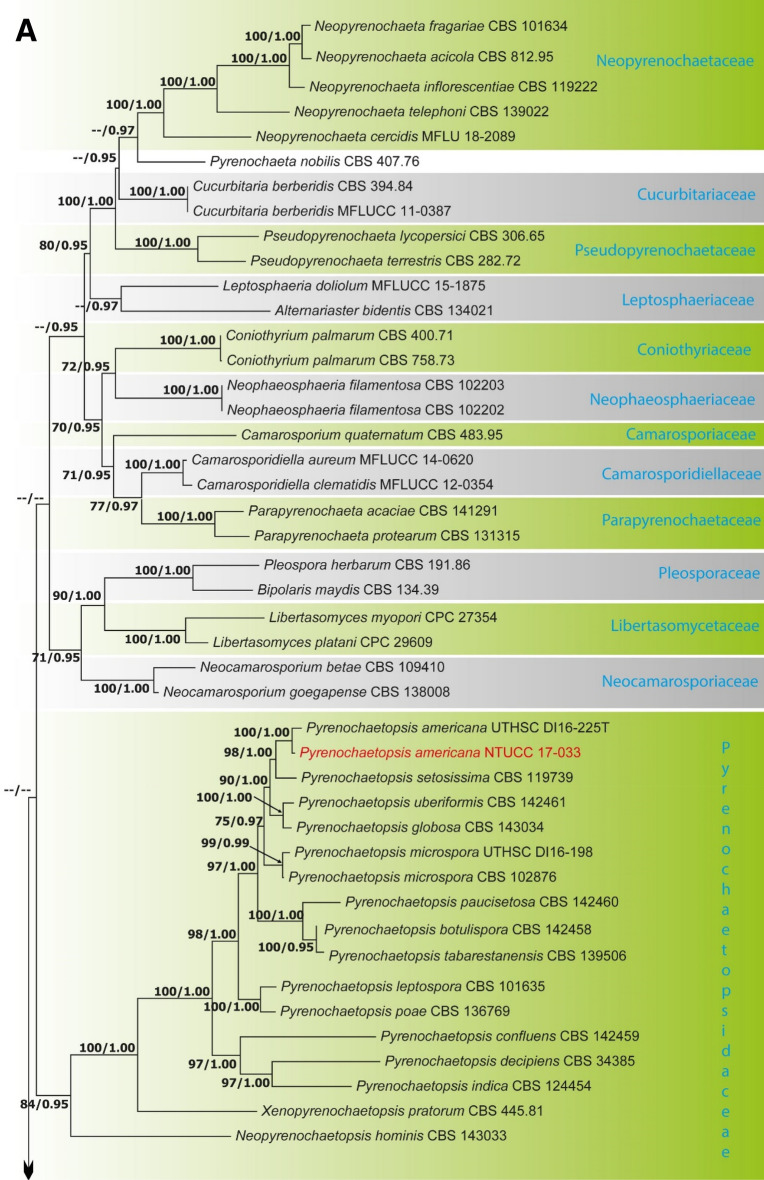

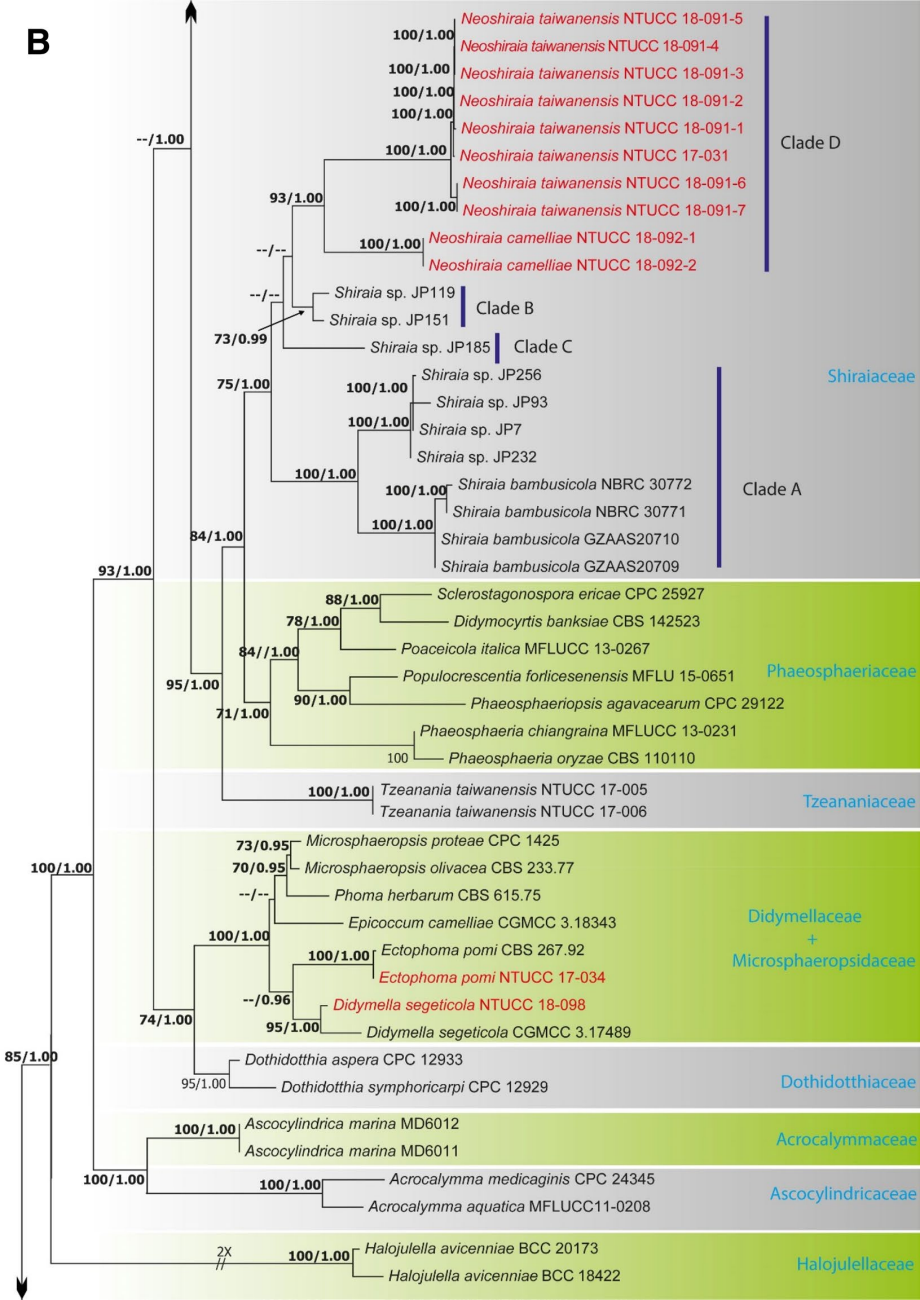

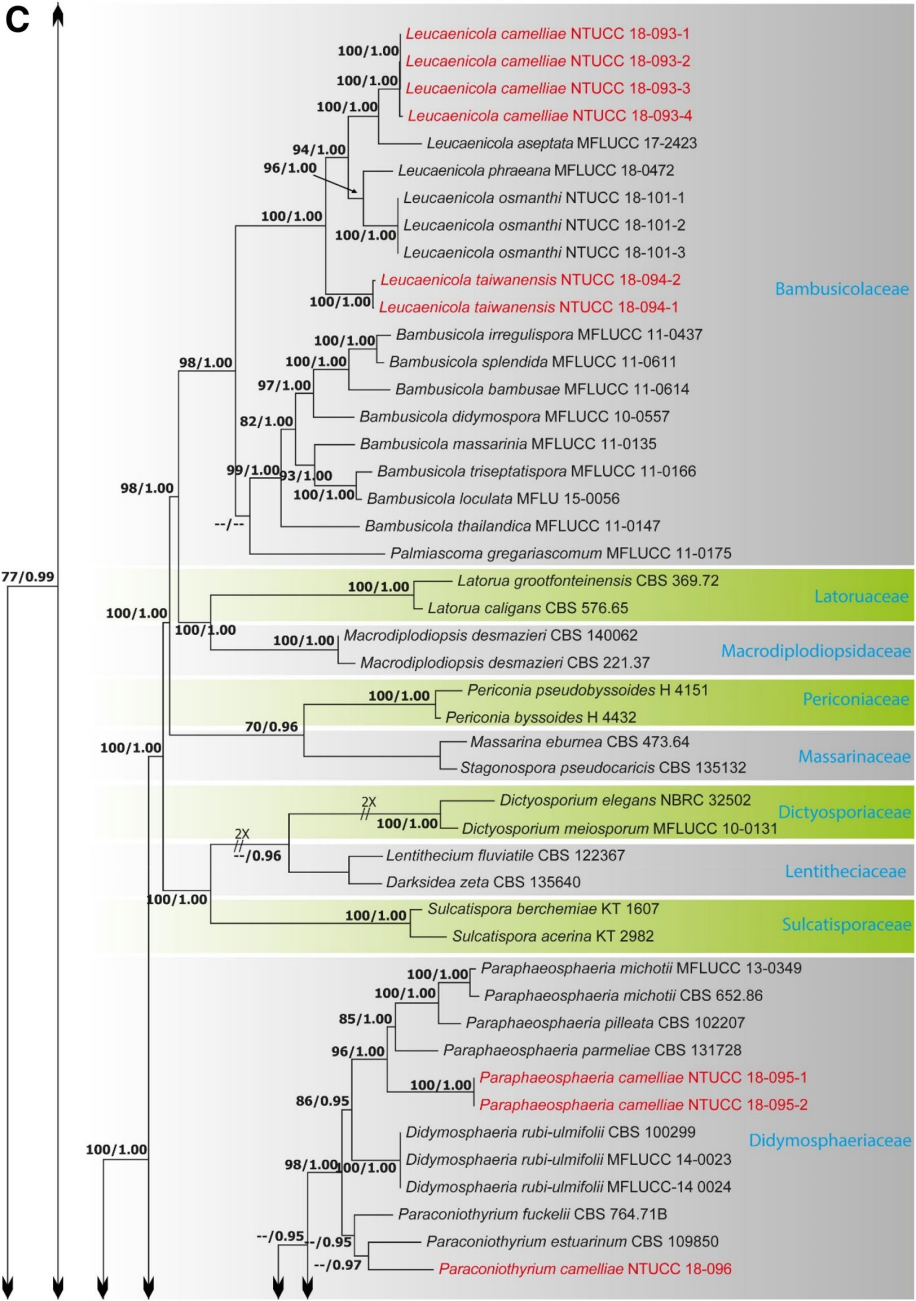

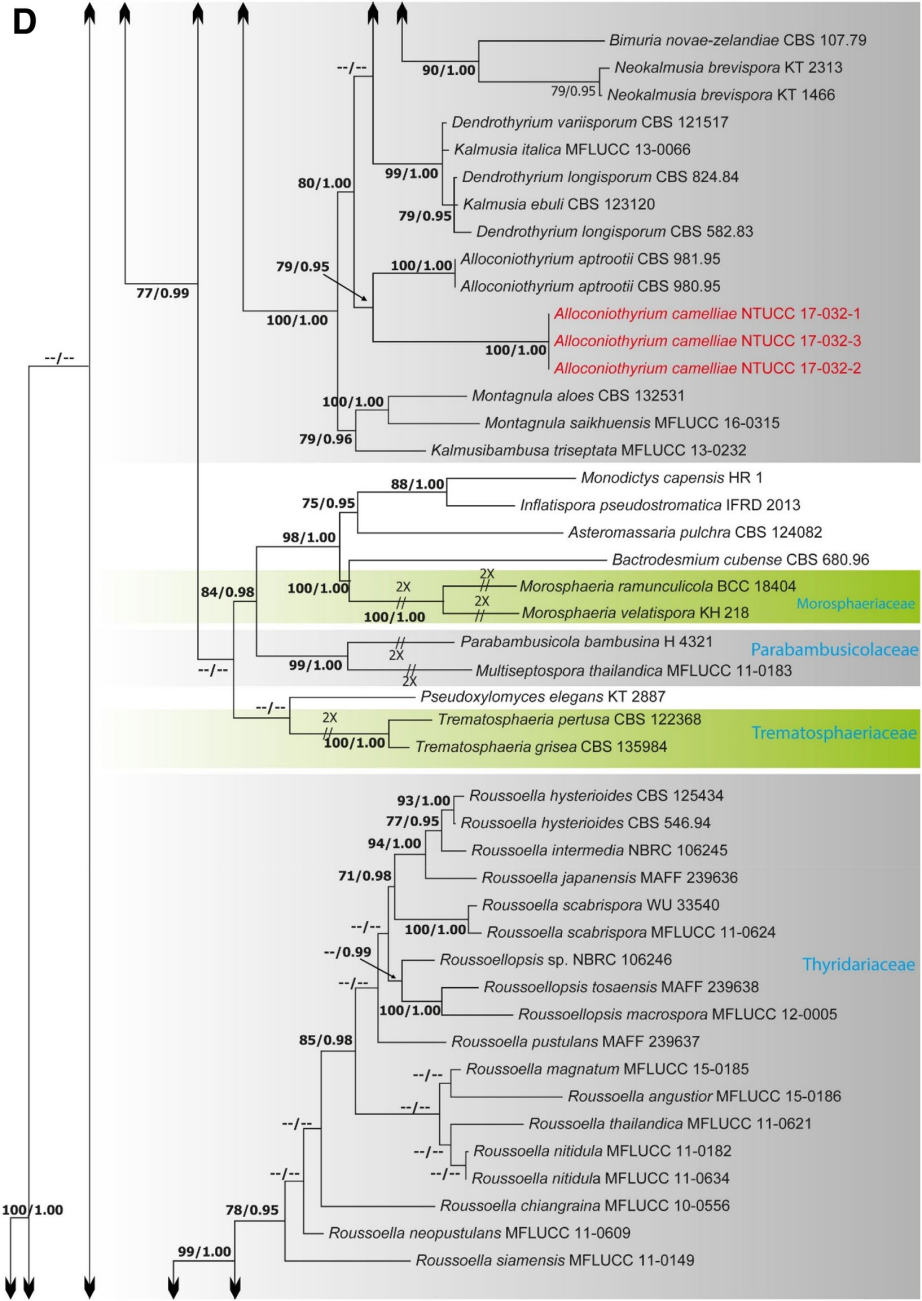

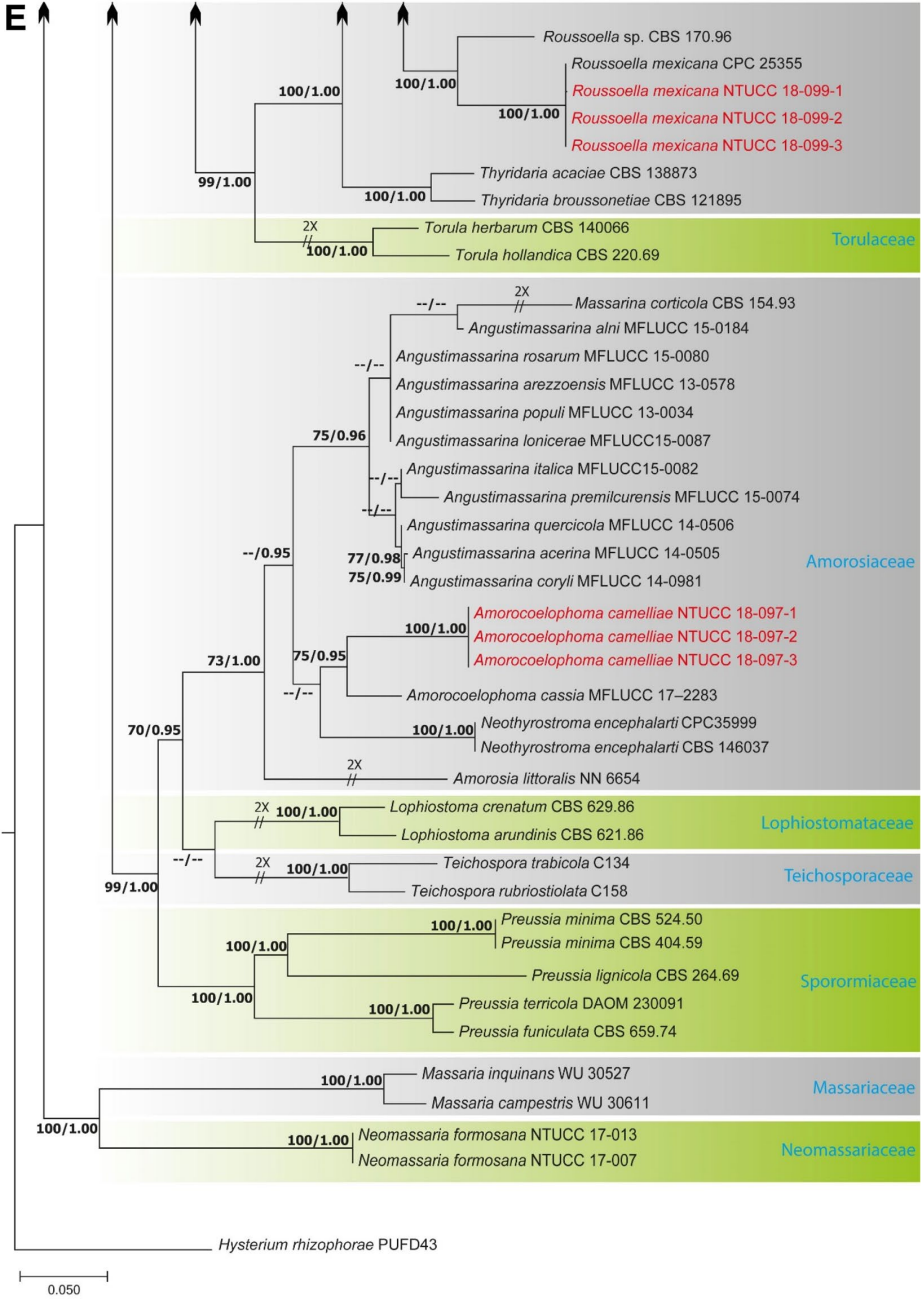
(**A**) Part one of the phylogenetic tree based on the concatenated alignment of six molecular markers (ITS, LSU, SSU, *rpb*2, *tef*1 and *tub*2) evaluated using RAxML. The new isolates are shown in red. ML bootstrap values (MLBS) ≥ 70% and Bayesian posterior probabilities (PP) ≥ 0.95 are presented at the nodes. The scale bar indicates the number of estimated substitutions per site. *Hysterium rhizophorae* (PUFD43) was used as an outgroup for rooting the tree. (**B**) Part two, (**C**) Part three, (**D**) Part four, (**E**) Part five of the phylogenetic tree based on the concatenated alignment of six molecular markers (ITS, LSU, SSU, *rpb*2, *tef*1 and *tub*2) evaluated using RAxML.

**Figure 2:**
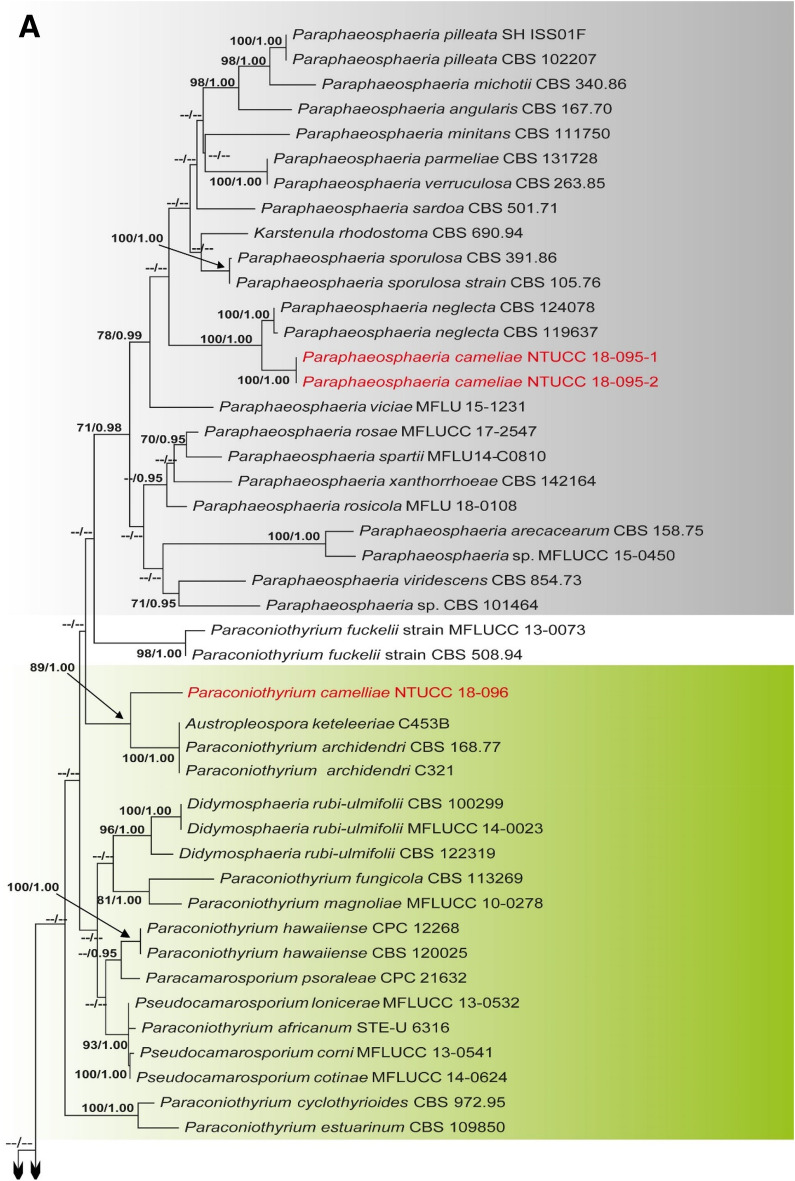

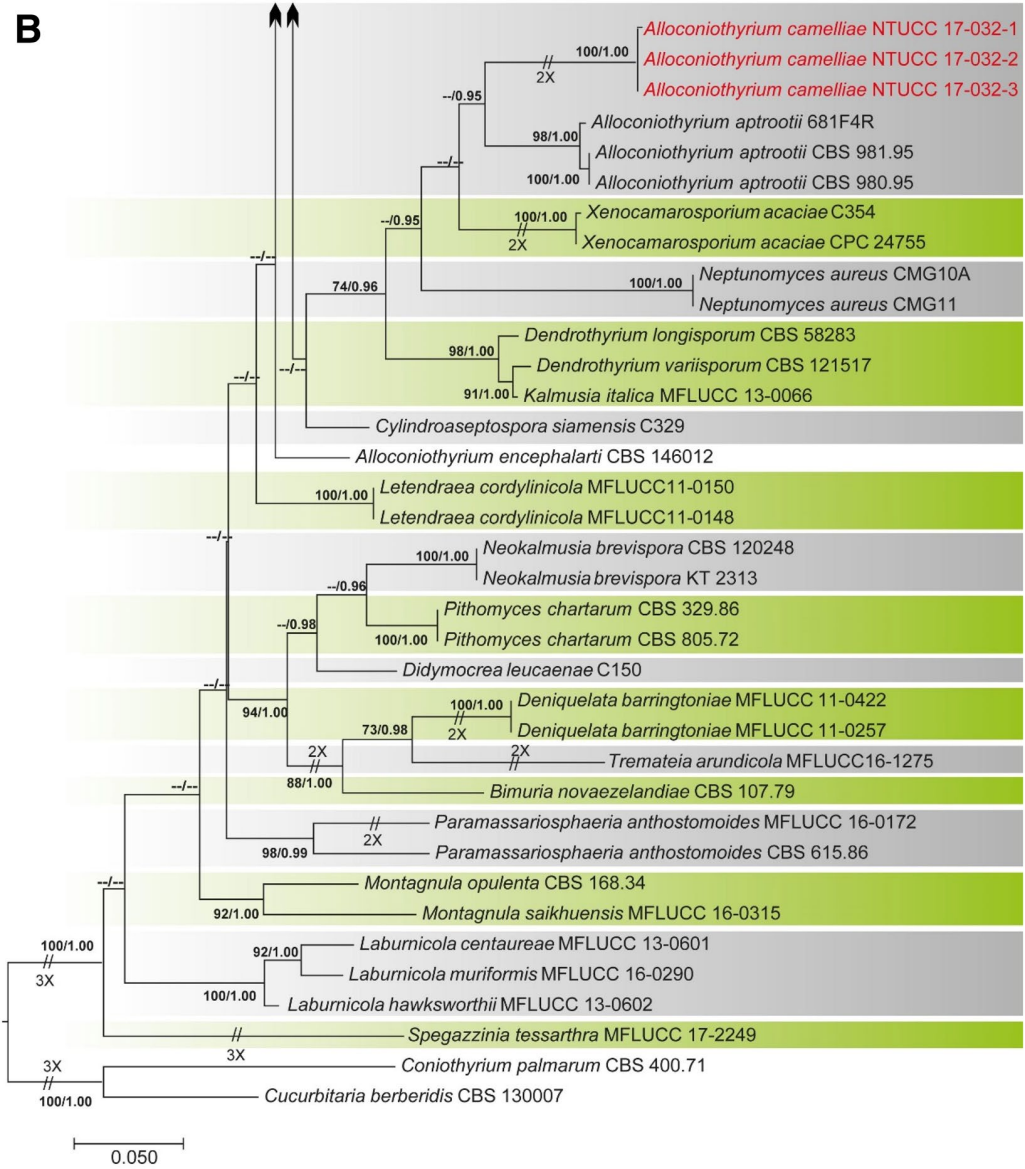
(**A**) Part one of the phylogenetic tree based on the concatenated alignment of four molecular markers (ITS, LSU, SSU, and *tub*2) evaluated using RAxML. The new isolates are shown in red. ML bootstrap values (MLBS) ≥ 70% and Bayesian posterior probabilities (PP) ≥ 0.95 are at each node. The scale bar indicates the number of estimated substitutions per site. *Cucurbitaria berberidis* (CBS 130007) and *Coniothyrium palmarum* (CBS 400.71) were used as outgroups for rooting the tree. (**B**) Part two of the phylogenetic tree based on the concatenated alignment of four molecular markers (ITS, LSU, SSU, and *tub*2) evaluated using RAxML.

Alignments corresponding to the single genes ITS, LSU, SSU, *rpb*2, *tef*1 and *tub*2 were analysed using the two phylogenies. Congruence between individual genes made it possible to reconstruct the phylogenies by concatenating the genes as it provided a guarantee of gene orthology (See Supplementary Data S1 and S2 for single gene trees). Phylogenetic trees obtained from the concatenated gene datasets are in Figs. 1 and 2. Comparison of the alignment properties and nucleotide substitution models are given in Supplementary Tables S2 and S3.

### Phylogeny of Pleosporales based on concatenated alignment of six molecular markers (ITS, LSU, SSU, *rpb2*, *tef*1 and *tub*2)

The dataset consisted of 5,229 characters (ITS 422, LSU 945, SSU 1,034, *rpb*2 1,080, *tef*1 938 and *tub*2 810). A best scoring RAxML tree is shown in Fig. 1, with the likelihood value of − 79,379.027274. The Bayesian analysis resulted in 90,000 trees after 90,000,000 generations. The first 18,000 trees, representing the burn-in phase of the analyses, were discarded, while the remaining trees were used for calculating Bayesian posterior probability (PP) in the majority rule consensus tree. All methods resulted in largely the same topology with high support for most branches in the ML and BI analyses and with similar overall topologies of family and genus level relationships in agreement with previous work based on ML and BI analyses^1–5,10,18,19^. A total of 229 strains representing 42 families including the 31 strains generated in the present study were included in the final Pleosporales alignment.

The family Shiraiaceae was resolved into four distinct clades (A, B, C and D). *Shiraia* (Clade A) includes the generic type of the genus, *S. bambusicola*, and putatively named *Shiraia* species strains JP256, JP93, JP7, and JP232; the new genus *Neoshiraia* (Clade D) with two species *N. camelliae* sp. nov and *N. formosanum* sp. nov. However, putatively named *Shiraia* species strains JP119, JP151 (Clade B) and JP185 (Clade C) formed two distinct clades basal to the *Neoshiraia* clade.

*Didymella segeticola* (NTUCCH 17-004) isolated in this study clustered in a well-supported clade with another isolate of *D. segeticola *(CGMCC 3.17498) that was used by Chen et al.^18^ to describe the species, therefore confirming the identification of the studied species. In addition, *Ectophoma pomi* (NTUCC 17-034) isolated from *C. sinensis* formed a highly supported clade with isolate CBS 267.92 of *E. pomi* that was examined by Valenzuela-Lopez et al.^5^ to describe the taxon, thus verifying the identification of the studied species. Furthermore, *Pyrenochaetopsis americana* (NTUCC 17-033) used in this study grouped in a well-supported clade with the type strain of *P. americana* (UTHSC D116-225T) that was used by Valenzuela-Lopez et al.^5^ to describe the species, hence validating the identification of the studied species.

The family Bambusicolaceae was composed of three clades, which correspond to the genera *Bambusicola*, *Leucaenicola* and *Palmiascoma*. For both ML and BI, and with both single locus and concatenated datasets, the four strains of *Leucaenicola* (NTUCC 18-093-1 to NTUCC 18-093-4) formed a distinct clade with high statistical support that was sister to the clade representing *L. aseptata* (MFLUCC 17-2423). Therefore, the new lineage is introduced here as a new species, *L. camelliae* sp. nov. Finally, the two strains of *Leucaenicola* (NTUCC 18-094-1 and NTUCC 18-094-2) isolated in the present study formed a basal terminal clade in *Leucaenicola* with both single locus and concatenated datasets. Thus, this new lineage is presented here as the new species *L. taiwanensis* sp. nov.

The family Thyridariaceae, which was highly supported in both ML and BI analyses, resulted in two clades that we identify as the genera *Thyridaria* and *Roussoella*. *R. mexicana* NTUCC 18-099-1, NTUCC 18-099-2 and NTUCC 18-099-3 in this study grouped in a well-supported clade with *R. mexicana* (CPC 25355) that was used by Crous et al.^20^ to introduce the species, therefore confirming the identification. The family Amorosiaceae grouped into four terminal clades, which correspond to known genera *Angustimassarina*, *Amorocoelophoma*, *Amorosia*, and *Neothyrostroma*. For both ML and BI, the three strains of *Amorocoelophoma* (NTUCC 18-097-1, NTUCC 18-097-2 and NTUCC 18-097-3) formed a distinct clade with high statistical support and sister to the clade representing *A. cassiae* (MFLUCC 17-2283) in both single locus and concatenated datasets. Thus, the novel lineage is presented here as *A. camelliae* sp. nov.

### Phylogeny of the family Didymosphaeriaceae based on the concatenated alignment of four molecular markers (ITS, LSU, SSU, and *tub*2)

The dataset in this analysis consisted of 2,970 characters (ITS 525, LSU 923, SSU 1,025, and *tub*2 497). The Bayesian analysis resulted in 10,000 trees after 10,000,000 generations. The first 20% of trees, representing the burn-in phase of the analysis, were discarded, while the remaining trees were used to calculate posterior probabilities in the majority rule consensus tree. A best scoring RAxML tree is shown in Fig. 2, with a likelihood of − 15,447.350059. All methods resulted in the same topology and had a similar overall topology for genus and species level relationships, consistent with former studies based on ML and BI analysis^3,5,18,19^.

The two strains of *Paraphaeosphaeria* NTUCC 18-095-1 and NTUCC 18-095-2 formed a distinct clade with high bootstrap support that is sister to the clade representing *Paraphaeosphaeria neglecta* (CBS 168.77 and MFLUCC 17-2429) in both single gene and concatenated gene analysis. Thus, the new lineage is recommended here as the new species *P. camelliae* sp. nov. Furthermore, the three strains of *Alloconiothyrium* (NTUCC 17-032-1, NTUCC 17-032-2 and NTUCC 17-032-3) form a separate clade with high statistical support that is sister to the clade representing *Alloconiothyrium aptrootii* (CBS 981. 95 and CBS 980. 95) in both single locus and multi locus analysis. Thus, the novel lineage is proposed here as the new species *A. camelliae* sp. nov*.*

The clade containing the single strain of *Paraconiothyrium* NTUCC 18-096 formed a separate clade with high statistical support that is sister to the clade representing *Paraconiothyrium archidendri* (CBS 168.77 and MFLUCC 17-2429) in both single locus and multi locus analysis. Thus, the novel lineage is proposed here as the new species *P. camelliae* sp. nov.

### Taxonomy

Based on multi-gene phylogenetic inference and morphological interpretations, numerous novel pleosporalean species were recognized in this study. These new taxa are described below.

#### Shiraiaceae

Y.X. Liu, Zi Y. Liu & K.D. Hyde, Phytotaxa 103: 53. 2013.

#### ***Neoshiraia***

Ariyawansa **gen. nov.**—MycoBank MB834500.

*Etymology* The name reveals the fact that species in this genus are comparable to, but dissimilar from those in the genus *Shiraia*.

Associated with leaf lesions of *C. sinensis*. *Sexual morph*: undetermined. *Asexual morph*: *Conidiomata* pycnidial, scattered over the surface of the leaf lesions, semi-immersed to erumpent, black, globose to subglobose, uni-loculate. *Conidiomatal wall* consisting of thick-walled cells of *textura angularis* that are dark brown to lightly-pigmented, and become hyaline towards the conidiogenous region. *Conidiophores* reduced to conidiogenous cells. *Conidiogenous cells* enteroblastic, phialidic, hyaline, ampulliform to doliiform or cylindrical, discrete or integrated. *Conidia* hyaline, aseptate, obovoid to ellipsoidal, smooth-walled.

*Type species* ***Neoshiraia camelliae*** Ariyawansa, I. Tsai & Thambugala **sp. nov.**

*Notes* Liu et al.^21^ established the family Shiraiaceae to accommodate the genus *Shiraia* Henn. Later, Morakotkarn et al.^22^ described multiple Shiraia-like strains, obtained from bamboo tissues as endophytes, that showed a close phylogenetic affinity to *S. bambusicola* (See Fig. 1B Clades A, B and C). The genus *Grandigallia* M.E. Barr et al., added later by Ariyawansa et al.^23^, differs from *Shiraia* in having black ascostromata and a *Polylepis* (Rosaceae) host.

A new genus is introduced here in Shiraiaceae to accommodate two coelomycetous species isolated from *C. sinensis* in Taiwan. *Shiraia* Henn. can be distinguished from *Neoshiraia* in having fusiform, muriform, asymmetrical, hyaline to light brown conidia whereas *Neoshiraia* possesses hyaline, aseptate, obovoid to ellipsoidal conidia. Moreover, Shiraia-like species are either parasitic or endophytic on bamboo, while *Neoshiraia* species are reported on leaf lesions of *C. sinensis* (tea).

We were unable to assign putatively named Shiraia-like species representing Groups A, B and C illustrated in Morakotkarn et al.^22^ to either *Shiraia* or *Neoshiraia* due to lack of phenotypic characters except DNA sequence evidence in these three groups. Morakotkarn et al.^22^, the authors who introduced these strains for the first time, reached a similar conclusion. Therefore, further examination of these fungi is necessary to elucidate the taxonomic placement of groups A, B and C in the family Shiraiaceae.

#### ***Neoshiraia camelliae***

**Figure 3:**
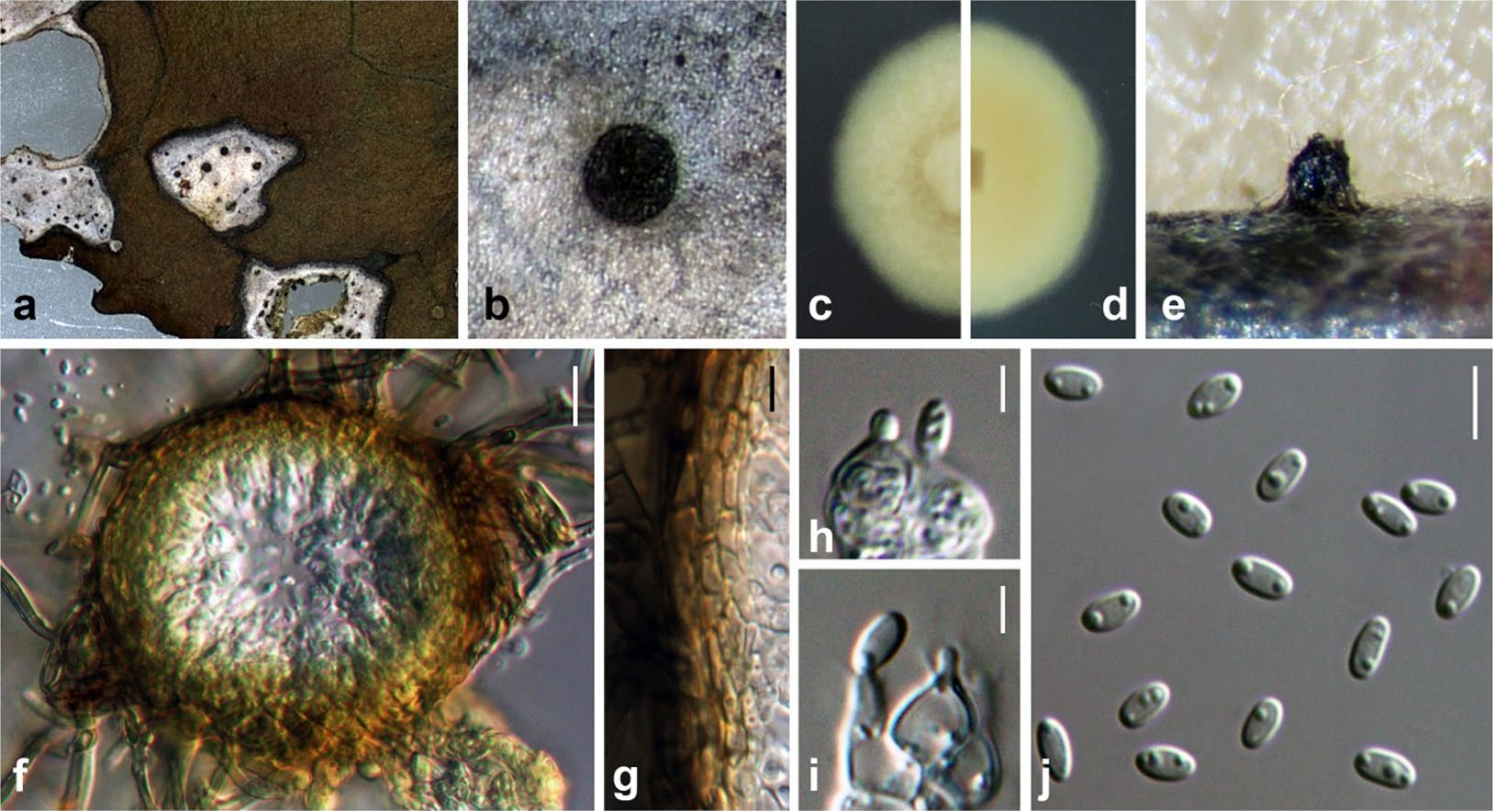
*Neoshiraia camelliae* (NTUH 18-092-1, **holotype**). (**a**) Leaf lesions. (**b**) Conidioma on host surface. (**c**, **d**) Cultures on PDA, from above and below. (**e**) Conidiomata on pine needle. (**f**) Vertical section through conidioma. (**g**) Conidiomatal wall. (**h**, **i**) Conidiogenous cells and developing conidia. (**j**) Conidia. Scale bars: (**f**) = 10 μm, (**g**, **j**) = 5 μm, (**h**, **i**) = 2.5 μm.

Ariyawansa, I. Tsai & Thambugala **sp. nov.**—MycoBank MB834501 Fig. 3.

*Etymology* Named after the host genus, *Camellia*, from which it was isolated.

Associated with leaf lesions of *C. sinensis*. *Leaf lesions* expanded, coalesced, and irregular. *Sexual morph*: undetermined. *Asexual morph*: *Conidiomata* pycnidial, scattered over the surface of the leaf lesions, semi-immersed to erumpent, black, globose to subglobose, uni-loculate. *Conidiomatal wall* consisting of dark brown to lightly-pigmented thick-walled cells of *textura angularis*, 4–7 × 1–3 μm (x̄ = 5.4 ± 1.0 × 2.1 ± 0.5 μm, n = 29). *Conidiophores* reduced to conidiogenous cells. *Conidiogenous cells* 5–7 × 3–4 μm (x̄ = 5.1 ± 0.7 × 4.0 ± 0.4 μm, n = 28), enteroblastic, phialidic, hyaline, ampulliform to doliiform, discrete or integrated, smooth. *Conidia* 4–5 × 2–3 μm (x̄ = 4.3 ± 0.2 × 2.5 ± 0.1 μm, n = 30), hyaline, aseptate, obovoid to ellipsoidal, guttulate, smooth-walled.

*Material examined*: Taiwan, Lugu Township, Nantou County, Feng-Huang Tea Fields, on leaves of *C. sinensis* (Theaceae), 15 April 2018, Tsai Ichen, ST06-1 (NTUH 18-092-1, **holotype**), ex-type culture ST06-1 (NTUCC 18-092-1); *ibid*., ST06-2 (NTUH 18-092-2), living culture ST06-2 (NTUCC 18-092-2).

#### ***Neoshiraia taiwanensis***

**Figure 4:**
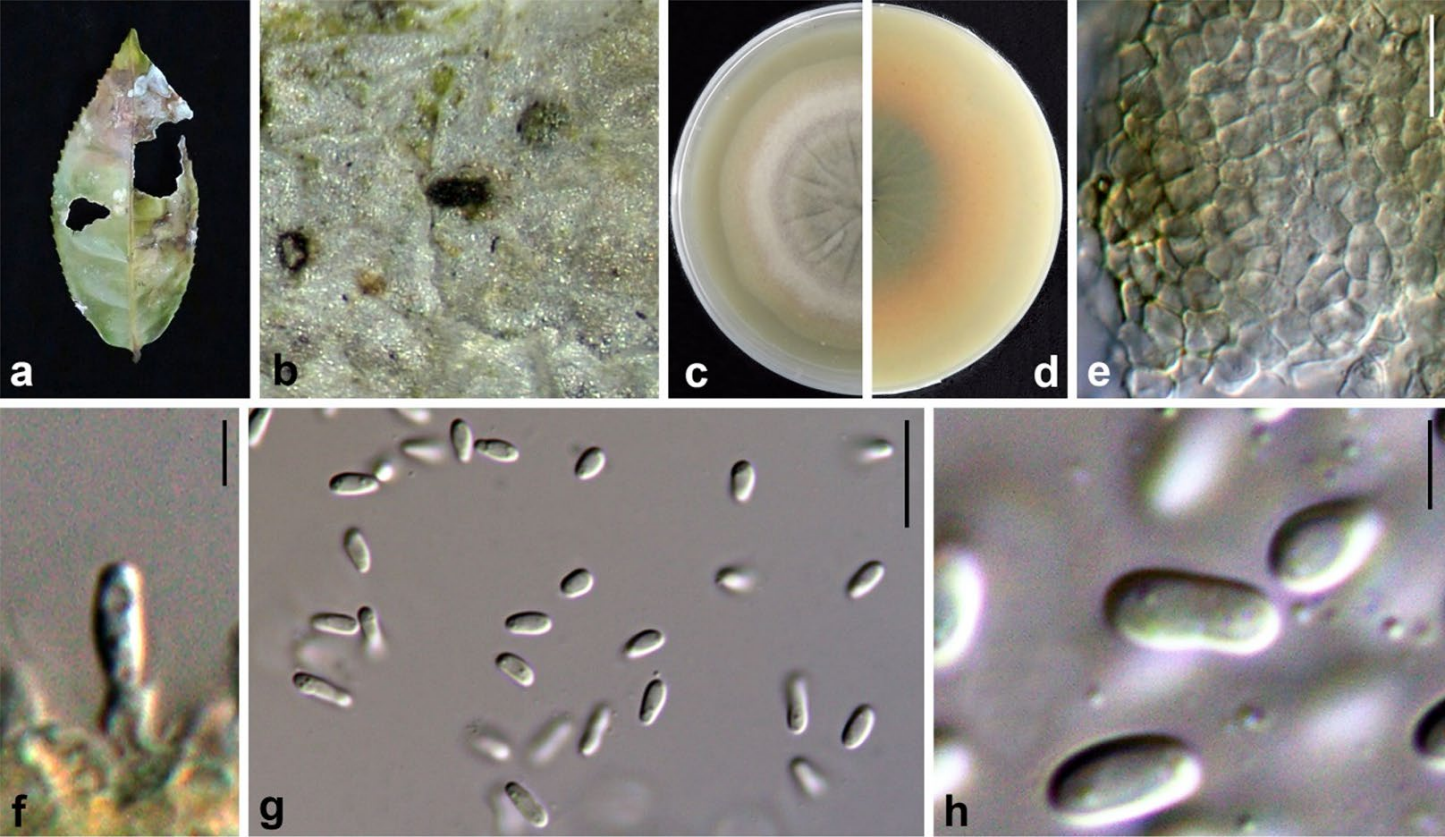
*Neoshiraia taiwanensis* (NTUH17-031, **holotype**). (**a**) Leaf lesions. (**b**) Conidioma on host surface. (**c**, **d**) Cultures on Emerson agar, from above and below. (**e**) Conidiomatal wall. (**f**) Conidiogenous cells and developing conidia. (**g**, **h**) Conidia. Scale bars: (**e**, **g**) = 10 μm, (**f**, **h**) = 2.5 μm.

Ariyawansa, I. Tsai & Thambugala **sp. nov.**—MycoBank MB834503 Fig. 4.

*Etymology* Named after the country, Taiwan, where the holotype was collected.

Associated with leaf lesions of *C. sinensis*. *Leaf lesions* expanded, coalesced, and developing from apex to middle of leaves. *Sexual morph*: undetermined. *Asexual morph*: *Conidiomata* pycnidial, scattered over the surface of the leaf lesions, immersed, slightly erumpent, black, globose to subglobose, uni-loculate. *Conidiomatal wall* consisting of dark brown to lightly pigmented thick-walled cells of *textura angularis*, becoming lightly pigmented to hyaline towards the conidiogenous region. *Conidiophores* reduced to conidiogenous cells. *Conidiogenous cells* 3–6 × 3–5 μm (x̄ = 4.7 ± 0.8 × 4.0 ± 0.6, n = 20) enteroblastic, phialidic, hyaline, ampulliform to doliiform or cylindrical, discrete or integrated, smooth. *Conidia* 4–5 × 2–2.5 μm (x̄ = 4.6 ± 0.4 × 2.0 ± 0.1 μm, n = 30), hyaline, aseptate, obovoid to ellipsoidal, guttulate, smooth-walled.

*Material examined* Taiwan, Shiding District, New Taipei City, Tea Research and Extension Station (Wenshan branch), on leaves of *C. sinensis* (Theaceae), 23 November 2017, Tsai Ichen, TR032 (NTUH 17-031, **holotype**), ex-type culture NTUCC 17-031; Hsinchu County, Hukou Township, on leaves of *C. sinensis* (Theaceae), 4 April 2018, Tsai Ichen, HK13 (NTUH 18-091-1), living culture HK13 (NTUCC 18-091-1); Yilan County, Datong Township, on leaves of *C. sinensis* (Theaceae), 1 June 2018, Tsai Ichen, YL04 (NTUH 18-091-2), living culture YL04 (NTUCC 18-091-2); *ibid*., YL07 (NTUH 18-091-3), living culture YL07 (NTUCC 18-091-3); Miaoli County, Tongluo Township, on leaves of *C. sinensis* (Theaceae), 18 May 2018, Tsai Ichen, TL03 (NTUH 18-091-4), living culture TL03 (NTUCC 18-091-4); Taitung County, Luye Township, on leaf of *C. sinensis* (Theaceae), 29 June 2018, Tsai Ichen, TT08 (NTUH 18-091-5), living culture TT08 (NTUCC 18-091-5); Yilan County, Jiaoxi Township, on leaf of *C. sinensis* (Theaceae), 3 April 2018, Tsai Ichen, JS01-6-1 (NTUH 18-091-6), living culture JS01-6-1 (NTUCC 18-091-6); *ibid*., JS01-6-2 (NTUH 18-091-7), living culture JS01-6-2 (NTUCC 18-091-7).

*Notes* The type species of *Neoshiraia*, *N*. *camelliae* is phylogenetically closely related to *N*. *taiwanensis* but has larger conidiogenous cells (5–7 × 3–4 μm vs 3–6 × 3–5 μm) and conidia (4–5 × 2–3 μm vs 4–5 × 2–2.5 μm).

#### Bambusicolaceae

D.Q. Dai & K.D. Hyde, Fungal Divers. 63: 49. 2013.

#### ***Leucaenicola***

Jayasiri, E.B.G. Jones & K.D. Hyde, Mycosphere 10: 37. 2019.

The genus *Leucaenicola* was introduced by Jayasiri et al.^24^ to place two coelomycetous species isolated from decaying pods of *Leucaena* sp. in Bambusicolaceae. *Leucaenicola* is characterized by pycnidial conidiomata with thin-walled conidiomatal wall comprising *textura angularis* or *textura prismatica* cells, enteroblastic, phialidic, hyaline to brown conidiogenous cells with one-celled, initially hyaline, becoming brown at maturity, oblong to ellipsoidal, aseptate, smooth-walled conidia.

#### ***Leucaenicola camelliae***

**Figure 5:**
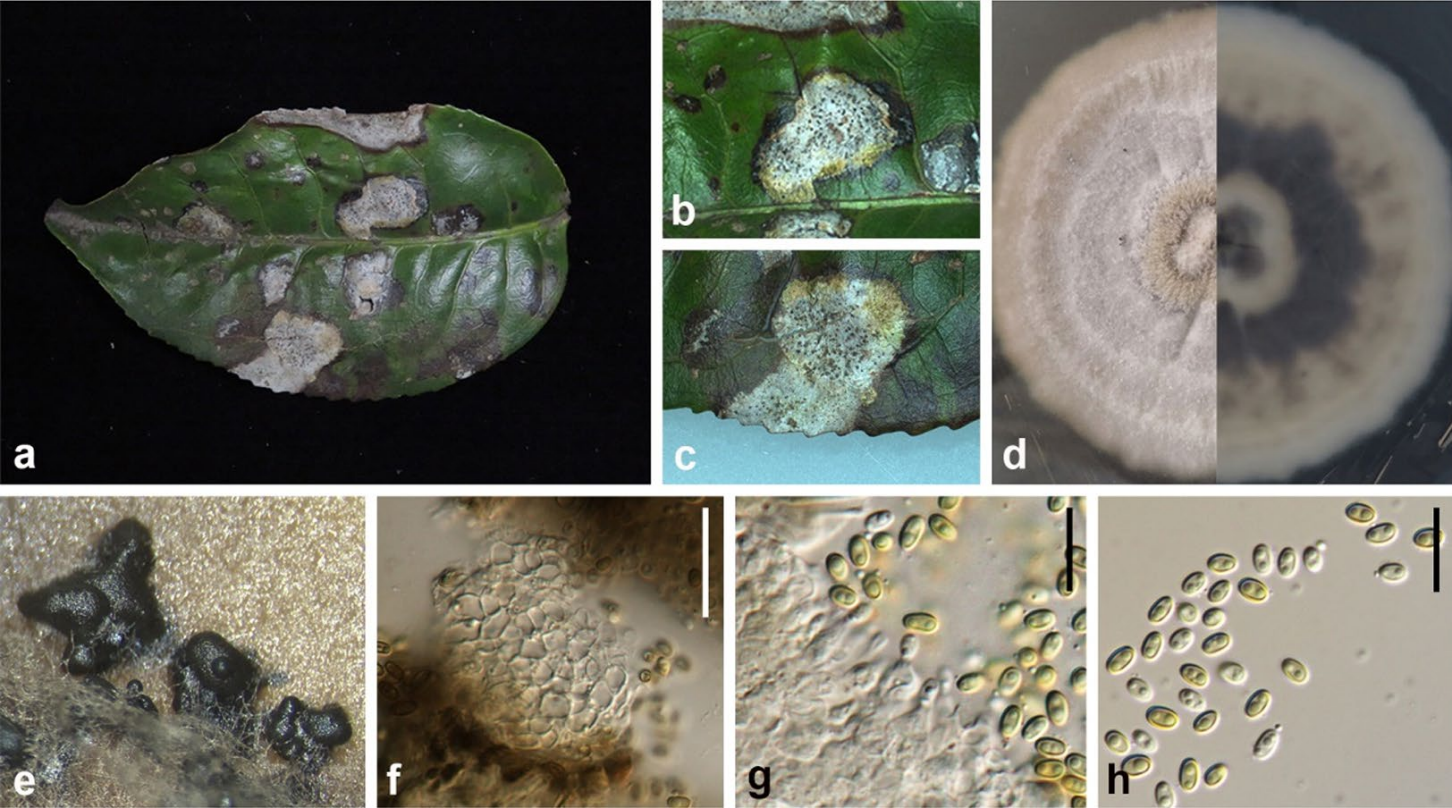
*Leucaenicola camelliae* (NTUH 18-093-4, **holotype**). (**a**) Leaf lesions. (**b**, **c**) Conidiomata on host surface. (**d**) Cultures on PDA, from above and below. (**e**) Conidiomata on PDA. (**f**) Conidiomatal wall. (**g**) Conidiogenous cells and developing conidia. (**h**) Conidia. Scale bars: (**f**) = 20 μm, (**g**, **h**) = 10 μm.

Ariyawansa, I. Tsai & Thambugala **sp. nov.**—MycoBank MB834504 Fig. 5.

*Etymology* Named after *Camellia*, the host genus from which it was isolated.

Associated with leaf lesions of *C. sinensis*. *Leaf lesions* expanded, coalesced, developing at edge and middle of leaves. *Sexual morph*: undetermined. *Asexual morph*: *Conidiomata* pycnidial, solitary or aggregated, scattered, immersed to slightly erumpent through the host tissues, uni-loculate, globose to subglobose. *Conidiomatal wall* 10–20 μm wide, comprising few layers of brown to hyaline thick-walled cells of *textura angularis* 3–6 × 2–4 μm (x̄ = 4.8 ± 0.7 × 3.1 ± 0.5 μm, n = 30). *Conidiophores* reduced to conidiogenous cells. *Conidiogenous cells* 3–6 × 2–3 μm (x̄ = 4.3 ± 0.6 × 3.0 ± 0.3 μm, n = 30), holoblastic, hyaline, smooth, ampulliform to doliiform, lining the conidiomatal cavity. *Conidia* 3–4 × 2–2 μm (x̄ = 4.0 ± 0.2 × 2.2 ± 0.1 μm, n = 30). ellipsoidal to cylindrical, initially hyaline, becoming pale brown, thin-walled, smooth, aseptate, with 1–2 guttules.

*Material examined*: Taiwan, Yangmei Distrct, Taoyuan City, Tea Research and Extension Station (Main), on leaves of *C. sinensis* (Theaceae), 29 January 2018, Tsai Ichen, TRM21-3 (NTUH 18-093-4, **holotype**), ex-type culture TRM21-3 (NTUCC 18-093-4); *ibid*., TRM09 (NTUH1-093-3), living culture TRM09 (NTUCC 18-093-3); Pingtung County, Manzhou Township, on leaves of *C. sinensis* (Theaceae), 26 May 2018, Tsai Ichen, SL16-1 (NTUH 18-093-1), living culture SL16A (NTUCC 18-093-1); *ibid*., SL16-2 (NTUH 18-093-2), living culture SL16B (NTUCC 18-093-2).

*Notes Leucaenicola camelliae* is phylogenetically closely related to *L*. *aseptata* and both species are similar in their general morphology. However, *L. camelliae* can be easily distinguished from *L*. *aseptata* in having a dark brown to black conidiomatal wall organized in a *textura angularis* to *textura prismatica*, phialidic, hyaline to brown, globose to flask-shaped conidiogenous cells plus the sizes of conidia. Furthermore, *L*. *camelliae* was reported on leaves of *C. sinensis* from Taiwan, while *L*. *aseptata* was found associated with decaying pods of *Leucaena* sp. (Fabaceae) from Thailand^24^.

#### ***Leucaenicola taiwanensis***

**Figure 6:**
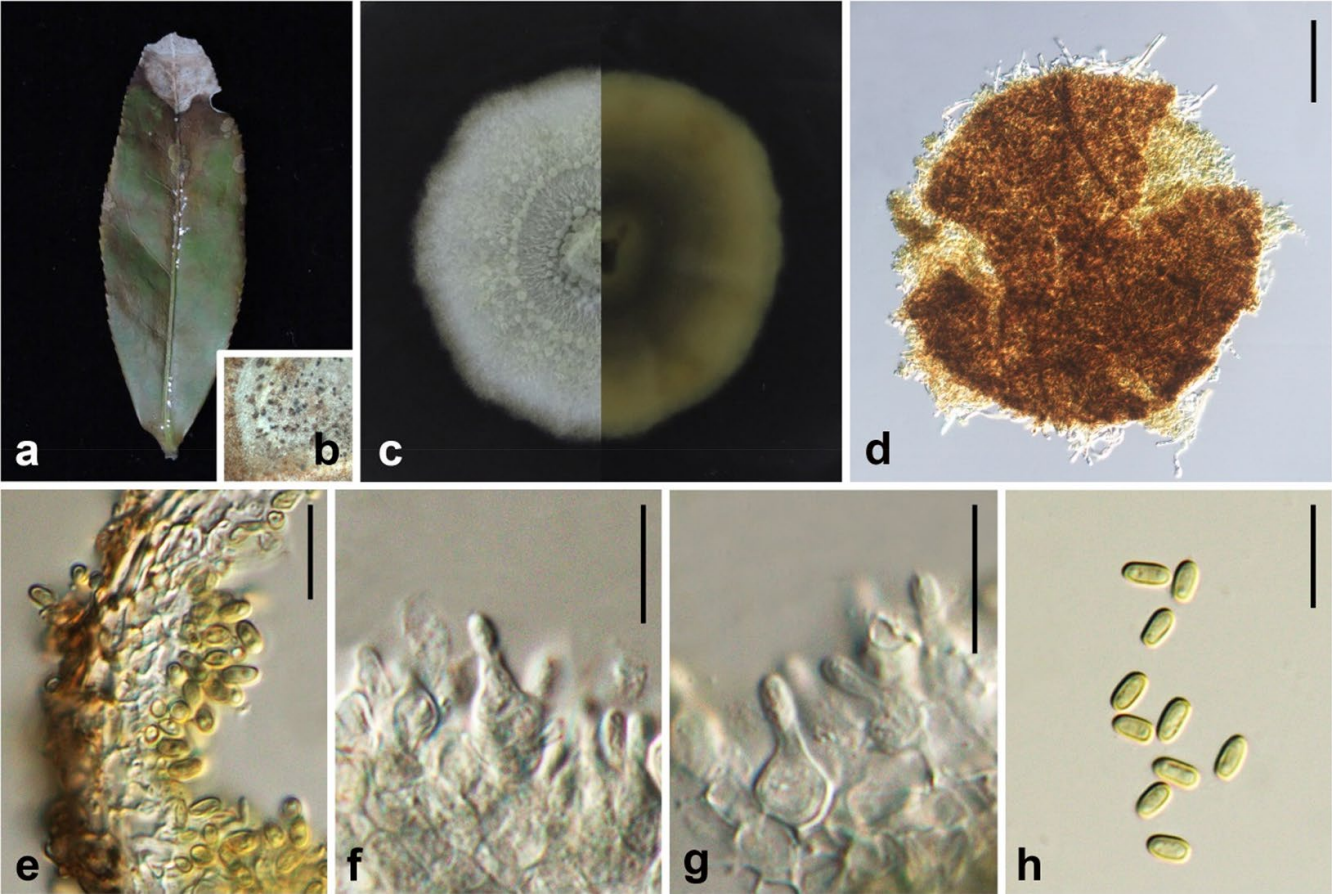
*Leucaenicola taiwanensis *(NTUH 18–094-1, **holotype**). (**a**) Leaf lesions. (**b**) Appearance of conidiomata on host surface. (**c**) Cultures on PDA, from above and below. (**d**) Pycnidium. (**e**) Conidiomatal wall. (**f**, **g**) Conidiogenous cells and developing conidia. (**h**) Conidia. Scale bars: (**d**) = 50 μm, (**e**–**h**) = 10 μm.

Ariyawansa, I. Tsai & Thambugala **sp. nov.**—MycoBank MB834507 Fig. 6.

*Etymology* Name refers to the country Taiwan, where it was collected.

Associated with leaf lesions of *C. sinensis*. *Leaf lesions* expanded, coalesced, developing at edge and apex of leaves. *Sexual morph*: undetermined. *Asexual morph*: *Conidiomata* pycnidial, solitary or aggregated, scattered, immersed to slightly erumpent through the host tissues, uni-loculate, globose to subglobose, ostiolate. *Conidiomatal wall* 10–20 μm wide, comprising few layers of dark brown to hyaline, thick-walled, cells of *textura angularis* to *textura prismatica*, 4–7 × 3–5 μm (x̄ = 5.7 ± 0.9 × 3.9 ± 0.5 μm, n = 30). *Conidiophores* reduced to conidiogenous cells. *Conidiogenous cells* 5–8 × 3–6 μm (x̄ = 6.3 ± 0.9 × 4.9 ± 0.9 μm, n = 30), holoblastic, hyaline, smooth, ampulliform to doliiform, lining the conidiomatal cavity. *Conidia* 4–6 × 2–3 μm (x̄ = 4.9 ± 0.3 × 2.5 ± 0.3 μm, n = 30), ellipsoidal to cylindrical, initially hyaline, becoming pale brown, smooth, aseptate, guttulate.

*Material examined* Taiwan, Yangmei District, Taoyuan City, Tea Research and Extension Station (Main), on leaves of *C. sinensis* (Theaceae), 29 January 2018, Ariyawansa Hiran, TRM08 (NTUH 18-094-1, **holotype**), ex-type culture TRM08 (NTUCC 18-094-1); *ibid*., TRM12-2 (NTUH 18-094-2), living culture TRM12-2 (NTUCC 18-094-2).

*Notes* *Leucaenicola taiwanensis* shares similar morphological characteristics with all other reported *Leucaenicola* species and mainly differs from them in having larger conidia. In the present phylogeny based on ITS, LSU, *rpb2*, *tef1* and *tub*2 sequences, *L. taiwanensis* formed a distinct clade basal to all other *Leucaenicola* species (Fig. 1C) in the family Bambusicolaceae.

#### ***Alloconiothyrium***

Verkley, Göker & Stielow, Persoonia 32: 33. 2014.

*Alloconiothyrium* was introduced by Verkley et al.^25^ as a monotypic genus for *A*. *aptrootii* which was isolated from a soil sample collected in Papua New Guinea. Based on morphology coupled with DNA sequence data, Verkley et al.^25^ places this new genus in Didymosphaeriaceae.

#### ***Alloconiothyrium camelliae***

**Figure 7:**
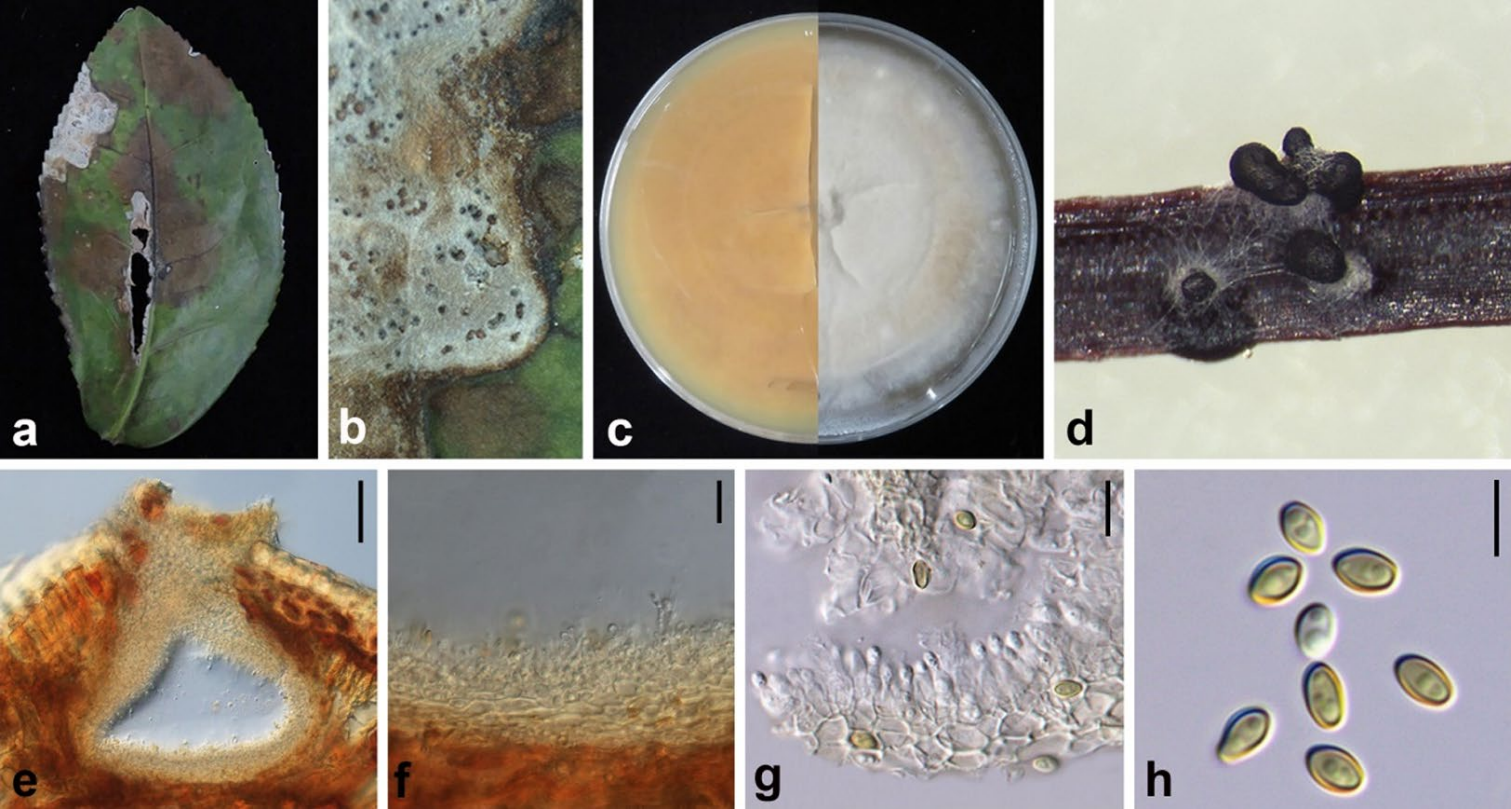
*Alloconiothyrium camelliae* (NTUH 17-032-1, **holotype**). (**a**) Leaf lesions. (**b**) Appearance of conidiomata on host surface. (**c**) Cultures on PDA, from above and below. (**d**) Pycnidia on pine needle. (**e**) Vertical section of conidioma. (**f**) Conidiomatal wall. (**g**) Conidiogenous cells and developing conidia. (**h**) Conidia. Scale bars: (**e**) = 50 μm, (**f**, **g**) = 10 μm, (**h**) = 5 μm.

Ariyawansa, I. Tsai & Thambugala **sp. nov.**—MycoBank MB834508 Fig. 7.

*Etymology* Name refers to the host genus *Camellia* from which it was isolated.

Associated with leaf lesions of *C. sinensis*. *Leaf lesions* grey, expanded, irregular, developing at edge and middle of leaves. *Sexual morph*: undetermined. *Asexual morph*: *Conidiomata* pycnidial, solitary or aggregated, scattered, immersed to slightly erumpent through the host tissues, uni-loculate, globose to subglobose. *Conidiomatal wall* 10–20 μm wide, comprising several layers of lightly pigmented to hyaline thick-walled, cells of *textura angularis* to *textura prismatica* 7–13 × 4–7 µm (x̄ = 9.6 ± 1.6 × 5.3 ± 0.9 µm, n = 30). *Conidiophores* reduced to conidiogenous cells. *Conidiogenous cells* 4–8 × 3–5 µm (x̄ = 5.9 ± 0.9 × 4.1 ± 0.6 µm, n = 30), holoblastic, hyaline, smooth, ampulliform to doliiform or cylindrical, lining the conidiomatal cavity. *Conidia*, 4–5 × 2–3 μm (x̄ = 4.3 ± 0.2 × 2.6 ± 0.1 μm, n = 30), ellipsoidal to cylindrical, initially hyaline, becoming pale brown, thin-walled, smooth, aseptate, guttulate.

*Material examined*: Taiwan, Shiding District, New Taipei City, Tea Research and Extension Station (Wenshan branch), on leaves of *C. sinensis* (Theaceae), 23 November 2017, Tsai Ichen, TR017-1-1 (NTUH 17-032-1, **holotype**), ex-type culture TR017-1-1 (NTUCC 17-032-1); *ibid*., TR017-1-2 (NTUH 17-032-2), living culture TR017-1-2 (NTUCC 17-032-2); *ibid*., TR017-1-3 (NTUH 17-032-3), living culture TR017-1-3 (NTUCC 17-032-3).

*Notes* In the present study we introduce another *Alloconiothyrium* species, *A*. *camelliae* from *C. sinensis* in Taiwan. *Alloconiothyrium aptrootii* can be distinguished from *A. camelliae* due to pycnidial or eustromatic conidiomata consisting of complexes with several cavities and covered by grey mycelium, discrete, broadly ampulliform, holoblastic, annellidic conidiogenous cells often with an elongated neck showing several distinct percurrent proliferations and verruculose conidia. In contrast, *A. camelliae* has uni-loculate, globose to subglobose conidiomata, ampulliform to doliiform or cylindrical conidiogenous cells and smooth-walled conidia^25,26^.

In a recent study Crous et al.^27^ introduced a species having coniothyrium-like morphology and cautiously classified it as *A. encephalarti*. However, in our multi-gene phylogeny, the type strain of *A. encephalarti* (CBS 146012) clustered in the family Didymosphaeriaceae but was separated from the generic clade of *Alloconiothyrium* and other genera of the family in a clade with relatively low statistical support (Fig. 2B). Therefore, generic placement of the tentatively named *A. encephalarti* strain in Didymosphaeriaceae remains unresolved. However, both *A. aptrootii* and *A. camelliae* can be easily distinguished from *A. encephalarti* by the shape and the sizes of the conidiogenous cells and conidia.

#### ***Paraphaeosphaeria***

O.E. Erikss., Ark. Bot. 6: 405. 1967.

The species of *Paraphaeosphaeria* produce coniothyrium-like asexual morphs characterized by eustromatic or pycnidial conidiomata, phialidic, or annelidic conidiogenous cells and brown, aseptate or 1-septate conidia. Recent studies have confirmed the placement of this genus in Didymosphaeriaceae^25,26,28^. It is important to perform phylogenetic analysis using sequence data from different gene loci to identify *Paraphaeosphaeria* species as it is very difficult to distinguish species of this genus based on morphology alone^25,26,28^. We have collected a new *Paraphaeosphaeria* species from tea leaves in Taiwan and confirmed its identity as a new species based on multi-locus phylogenetic analyses (Fig. 2A). This is the first record of *Paraphaeosphaeria* species from *C. sinensis* in Taiwan.

#### ***Paraphaeosphaeria camelliae***

**Figure 8:**
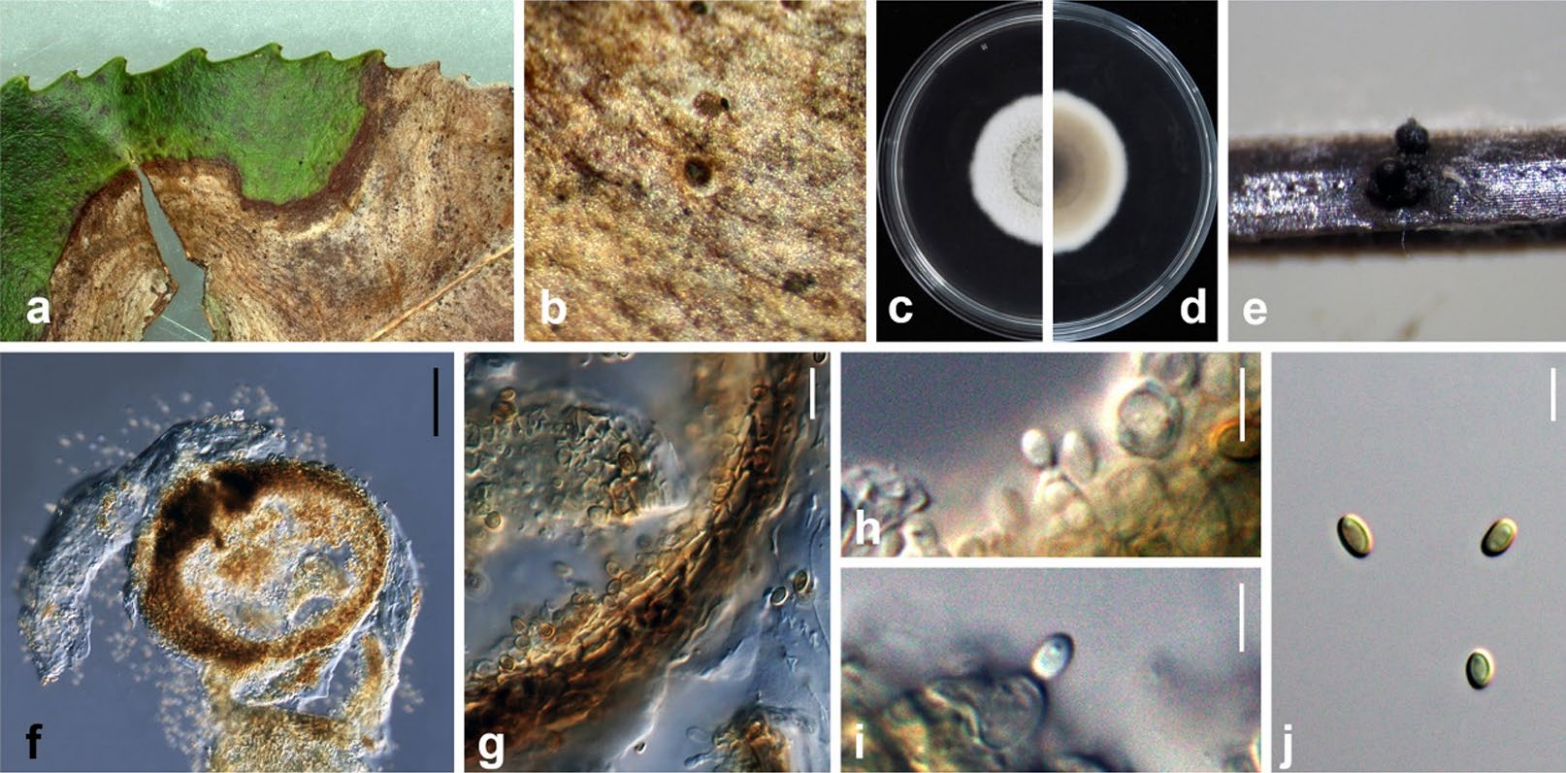
*Paraphaeosphaeria camelliae* (NTUH 18-095-2, **holotype**). (**a**) Leaf lesions. (**b**) Appearance of conidiomata on host surface. (**c**, **d**) Cultures on PDA, from above and below. (**e**) Pycnidia on pine needle. (**f**) Vertical section of conidioma. (**g**) Conidiomatal wall. (**h**, **i**) Conidiogenous cells and developing conidia. (**j**) Conidia. Scale bars: (**f**) = 50 μm, (**g**) = 10 μm, (**h**–**j**) = 5 μm.

Ariyawansa, I. Tsai & Thambugala **sp**. **nov**.—MycoBank MB834509 Fig. 8.

*Etymology* Name refers to the host genus *Camellia* from which it was isolated.

Associated with leaf lesions of *C. sinensis*. *Leaf lesions* brownish, expanded and developing from apex to middle of leaves. *Sexual morph*: undetermined. *Asexual morph*: *Conidiomata* pycnidial, solitary, scattered, immersed to slightly erumpent, uni-loculate, subglobose, ostiolate. *Conidiomatal wall* 10–15 μm wide, comprising several layers of dark brown to lightly pigmented thick-walled cells of *textura angularis*, 4–9 × 2–5 µm (x̄ = 5.8 ± 1.4 × 2.8 ± 0.7 µm, n = 28). *Conidiophores* reduced to conidiogenous cells. *Conidiogenous cells* 4–9 × 3–6 µm (x̄ = 6.1 ± 1.1 × 4.3 ± 0.9 µm, n = 30), holoblastic, hyaline, smooth, ampulliform to doliiform, lining the conidiomatal cavity. *Conidia* 3–5 × 2–4 µm (x̄ = 3.9 ± 0.4 × 2.9 ± 0.3 µm, n = 30), ellipsoidal to cylindrical, initially hyaline, becoming pale brown, thin-walled, smooth, aseptate, with 1–2 small guttules.

*Material examined* Taiwan, Fanlu Township, Chiayi County, Shengli Farm, on leaves of *C. sinensis* (Theaceae), 22 September 2018, Tsai Ichen, AM07-2 (NTUH 18-095-2, **holotype**), ex-type culture AM07-2 (NTUCC 18-095-2); *ibid*., AM01-2 (NTUH 18-095-1), living culture AM01-2 (NTUCC 18-095-1).

*Notes* *Paraphaeosphaeria camelliae* has a conidial morphology that is the same as *P. neglecta*, demonstrating that these two taxa are cryptic species. At present, the species identification of cryptic taxa are mainly resolved by phylogenies based on multi locus sequence data coupled with ecology (containing host range and pathogenicity), distribution or physiology (Crous et al.^20^). Other than the difference in phylogenetic inference, *P. camelliae* differs from *P. neglecta* by pathogenicity (pathogenic vs saprobic), host (*C. sinensis* vs *Cassia* sp.) and distribution (Taiwan vs Chile) ^25^.

#### ***Amorocoelophoma***

Jayasiri, E.B.G. Jones & K.D. Hyde, Mycosphere 10: 25. 2019.

Jayasiri et al.^24^ established a novel coelomycetous genus, *Amorocoelophoma* in Amorosiaceae Thambug. & K.D. Hyde to accommodate *A*. *cassia* from fallen pods of *Cassia* sp. (Fabaceae). In the present study, an *Amorocoelophoma* species was collected from leaves of *C. sinensis* (Theaceae) and identified as a new species (*A. camelliae*) based on its morphological characters and DNA sequence analyses.

#### ***Amorocoelophoma camelliae***

**Figure 9:**
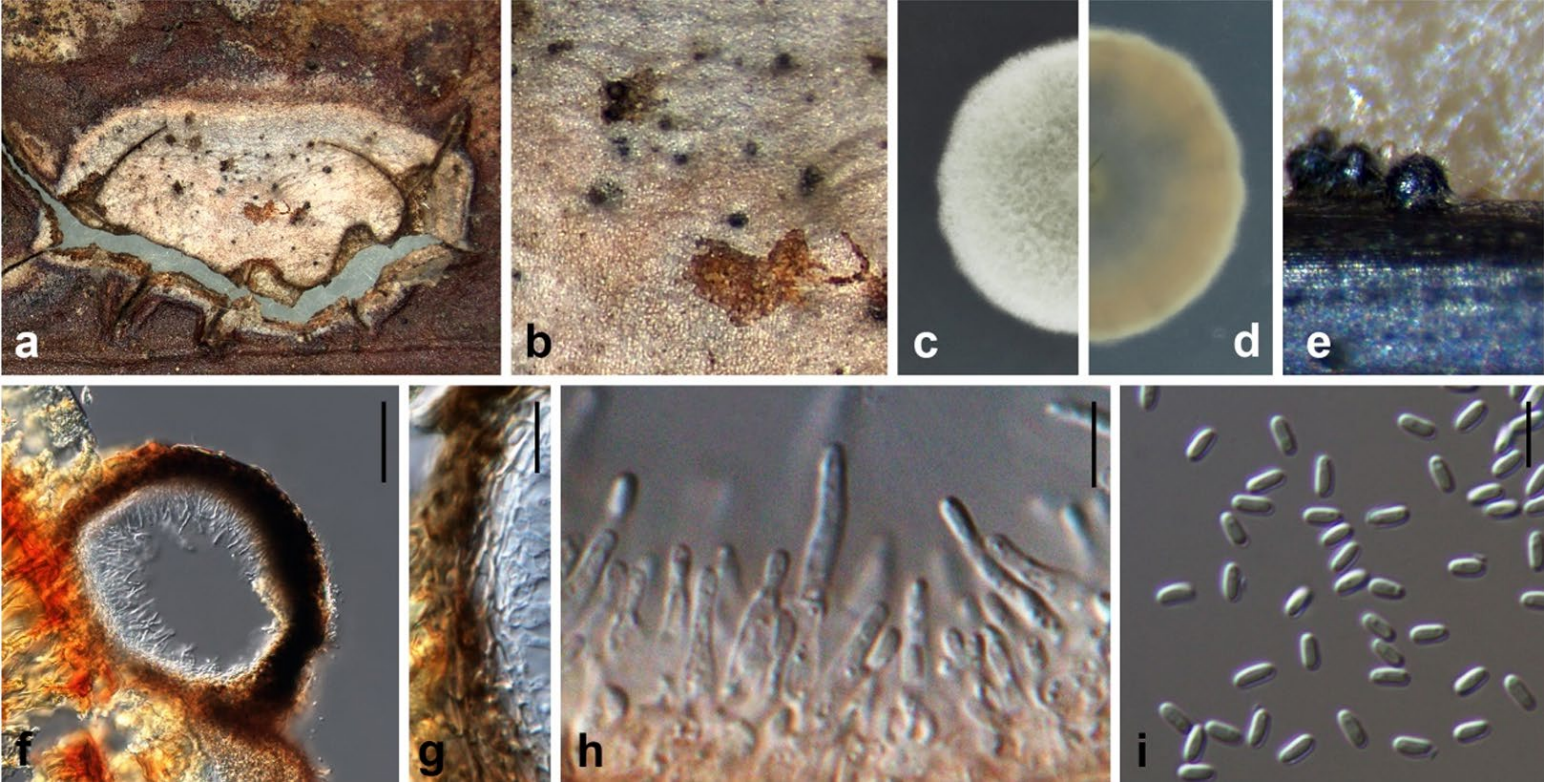
*Amorocoelophoma camelliae* (NTUH 18-097-1, **holotype**). (**a**) Leaf lesions. (**b**) Appearance of conidiomata on host surface. (**c**, **d**) Cultures on PDA, from above and below. (**e**) Pycnidia on pine needle. (**f**) Vertical section of conidioma. (**g**) Conidiomatal wall. (**h**) Conidiogenous cells and developing conidia. (**i**) Conidia. Scale bars: (**f**) = 50 μm, (**g**) = 10 μm, (**h**, **i**) = 5 μm.

Ariyawansa, I. Tsai & Thambugala **sp. nov.**—MycoBank MB834510 Fig. 9.

*Etymology* Name denotes the host, *Camellia*, from which it was collected.

Associated with leaf lesions of *C. sinensis*. *Leaf lesions* grey, expanded and coalesced. *Sexual morph*: undetermined. *Asexual morph*: *Conidiomata* pycnidial, solitary, scattered, immersed to slightly erumpent, uni-loculate, subglobose, ostiolate. *Conidiomatal wall* 20–26 μm wide, comprising few layers of dark brown to hyaline thick-walled cells of *textura angularis*, 3–9 × 2–3 µm (x̄ = 5.6 ± 1.7 × 2.5 ± 0.5 µm, n = 29). *Conidiophores* reduced to conidiogenous cells. *Conidiogenous cells* 3–10 × 2–4 µm (x̄ = 6.3 ± 1.8 × 2.1 ± 0.5 µm, n = 30), holoblastic, hyaline, smooth, ampulliform to cylindrical, lining the conidiomatal cavity. *Conidia* 2–3 × 1–2 µm (x̄ = 2.8 ± 0.2 × 1.5 ± 0.2 µm, n = 30), ellipsoidal to cylindrical, hyaline, thin-walled, smooth, aseptate.

*Material examined* Taiwan, Manzhou Township, Pintung County, Lao-Shan Tea Store, on leaves of *C. sinensis* (Theaceae), 26 May 2018, Tsai Ichen, SL13-1 (NTUH 18-097-1, **holotype**), ex-type culture SL13-1 (NTUCC 18-097-1); *ibid*., SL13-2 (NTUH 18-097-2), living culture SL13-2 (NTUCC 18-097-2); *ibid*., SL13-3 (NTUH 18-097-3), living culture SL13-3 (NTUCC 18-097-3).

*Notes* Morphologically *A. camelliae* is similar to *A. cassiae* but differs in its smaller conidia (2–3 × 1–2 µm vs 9–11 × 2–3 μm), host (*C. sinensis* vs *Cassia* sp.) and distribution (Taiwan vs Thailand)^24^.

#### ***Paraconiothyrium***

Verkley, Stud. Mycol. 50: 327. 2004.

This genus includes plant pathogenic, saprobic and endophytic species associated with a wide range of hosts and substrates worldwide^26,29^. *Paraconiothyrium* species appear to be paraphyletic within the family Didymosphaeriaceae^26^. Currently 24 species are recognized in the genus and they have been separated on the basis of morphological and molecular characterization. In some cases, multi-gene sequence data are necessary to unambiguously identify the species. *Paraconiothyrium camelliae* is introduced here as a novel species associated with leaf lesions of *C. sinensis*.

#### ***Paraconiothyrium camelliae***

**Figure 10:**
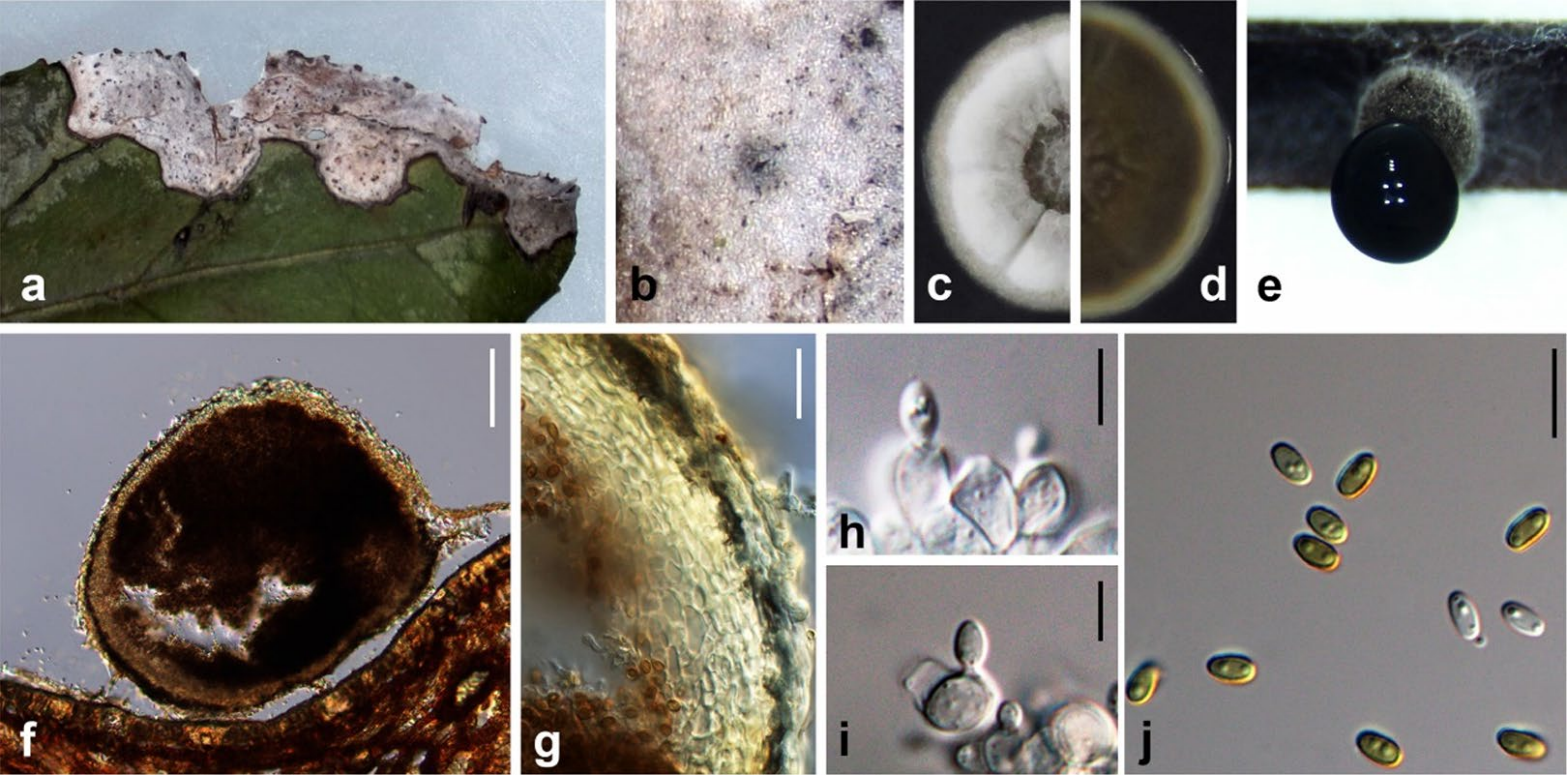
*Paraconiothyrium camelliae* (NTUH 18–096, **holotype**). (**a**) Leaf lesions (**b**) Appearance of conidiomata on host surface. (**c**, **d**) Cultures on PDA, from above and below. (**e**) Pycnidia on pine needle. (**f**) Vertical section of conidioma. (**g**) Conidiomatal wall. (**h**, **i**) Conidiogenous cells and developing conidia. (**j**) Conidia. Scale bars: (**f**) = 100 μm, (**g**) = 20 μm, (**h**, **i**) = 5 μm, (**j**) = 10 μm.

Ariyawansa, I. Tsai & Thambugala **sp. nov.**—MycoBank MB834511 Fig. 10.

*Etymology* Name refers to the host genus *Camellia* from which it was isolated.

Associated with leaf lesions of *C. sinensis*. *Leaf lesions* grey, expanded, irregular and developing from edge to middle of leaves. *Sexual morph*: undetermined. *Asexual morph*: *Conidiomata* pycnidial, solitary, scattered, immersed to uni-loculate, globose to subglobose, ostiolate. *Conidiomatal wall* 20–30 μm wide, comprising several layers of black to lightly pigmented thick-walled cells of *textura angularis*, 4–12 × 3–8 µm (x̄ = 6.3 ± 2.2 × 4.0 ± 1.1 µm, n = 30), becoming flattened and highly pigmented, towards the outer region of the conidiomata. *Conidiophores* reduced to conidiogenous cells. *Conidiogenous cells* 5–8 × 4–6 µm (x̄ = 5.8 ± 0.7 × 4.7 ± 0.6 µm, n = 30), holoblastic, hyaline, smooth, ampulliform to doliiform, lining the conidiomatal cavity. *Conidia* 4–6 × 2–3.5 µm (x̄ = 5.1 ± 0.3 × 3.1 ± 0.2 µm), ellipsoidal to cylindrical, initially hyaline, becoming pale brown, thin-walled, smooth, aseptate, with 1–2 small guttules.

*Material examined* Taiwan, Yuchi Township, Nantou County, Tea Research and Extention Station (Yuchi branch), on leaves of *C. sinensis* (Theaceae), 13 April 2018, Tsai Ichen, YC04 (NTUH 18-096, **holotype**), ex-type culture YC04 (NTUCC 18-096).

*Notes* *Paraconiothyrium camelliae* resides in a distinct clade sister to *P*. *archidendri* (Fig. 2A). However, *P. camelliae* differs morphologically from *P*. *archidendri* in having larger conidiogenous cells (5–8 × 4–6 µm vs 3.5–5 × 2.5–4 µm) and relatively bigger conidia (4–6 × 2–3.5 µm vs 3.5–6 × 2.5–3.5 (µm). Furthermore, *P*. *archidendri* was isolated from the leaf spots of *Archidendron bigeminum* in Burma, whereas *P*. *camelliae* was isolated from the diseased leaves of *C. sinensis* in Taiwan. *Paraconiothyrium camelliae* differs from the generic type of *Paraconiothyrium, P*. *estuarinum*, in having larger conidiogenous cells (5–8 × 4–6 µm vs 4–6.5 × 2.5–3.5 µm) and larger conidia (4–6 × 2–3.5 µm vs 3.2–4 × 1.4–1.7 µm) ^29^. Moreover, *P*. *estuarinum* was isolated from an estuarine sediment polluted with industrial discharges from Brazil, while *P*. *camelliae* was reported on leaves of *C. sinensis* from Taiwan.

## Discussion

Recently, large number of fungal species have been identified through phylogenetic studies based on DNA sequence data. These studies have helped scientists to understand the cryptic nature of many pathogenic fungal groups classified in the order Pleosporales such as *Alternaria*^10,30^, *Phoma*^25^, *Coniothyrium*^25^, *Paraconiothyrium*^25,29^ and *Paraphaeosphaeria*^25,26,29^. Furthermore, these studies suggest that multi-gene phylogenies together with phenotypic features are necessary to identify the species/genus of fungal pathogens classified in Pleosporales^10,25,30^.

Fungal disease development and weather conditions are highly correlated and this relationship is regularly accounted for in disease management and forecasting^31^. However, disease severity and the epidemiology of fungal pathogens can vary over time due to climatic change^31^. In recent years, the general climate patterns have been drastically changed mainly due to global warming, resulting in major effects on natural ecosystems^32^. As a direct consequences of this scenario, threats from unpredicted incidences of plant diseases have increased^33–35^.

*Camellia sinensis* (tea) is an evergreen shrub that is widely cultivated throughout tropical and subtropical regions of Asia and Africa. Tea is reported to have a wide range of beneficial physiological and medicinal effects^36–39^. Several fungal pathogens and endophytes are associated with the tea plant and fungal pathogens cause a significant threat to tea leaves^40–42^. Brown blight and leaf blotch caused by *Colletotrichum* species, grey blight caused by pestalotiopsis-like species, blister blight (*Exobasidium vexans* Massee), twig die-back and stem canker (*Macrophoma theicola* Petch) are common fungal diseases affecting tea plantations in major tea growing countries^12^. However, the global species diversity of Pleosporalean taxa associated with *C. sinensis* have received limited attention.

In the present study, we identified one genus and eight species associated with leaf spots of *C. sinensis* in Taiwan that are new to science. Apart from the novel species introduced, *D. segeticola*, *E. pomi* and *Roussoella mexican* were identified for the first time as foliar pathogens of *C. sinensis*. Our results can be used to develop a system to characterize and identify pleosporalean species in common practice and discover potential bio-control agents either to cure or minimize the damage done by phytopathogenic pleosporalean taxa linked with tea leaves in Taiwan.

## Materials and methods

### Sample collection and isolation

The field survey was carried out in seven organic and three commercial tea fields, and three tea research stations (Nov. 2017 to Sep. 2018) in the nine main tea cultivation provinces (Taipei, New Taipei, Taoyuan, Yilan, Hsinchu, Nantou, Chiayi, Hualien, Pingtung and Miaoli) in Taiwan. Diagnostic samples were obtained from infected tealeaves displaying leaf spots, characterized by tiny black spots of less than 1 mm diam., accompanied by chlorosis and browning, and resulting in defoliation. A single conidium isolation was done following the method described in Ariyawansa et al.^43^ in plant tissues where spore masses were formed (diseased leaves) that were obtained from the tea fields.

### Morphological examination

Morphological descriptions were made from isolates cultured on 2% potato dextrose agar (PDA; DIFCO) following the method described in Ariyawansa et al.^2^. Preparations for microscopy were made in distilled water, checked with an Olympus BX51 microscope with differential interference contrast (DIC) illumination and at least 30 measurements per structure were noted^2^. Voucher specimens were deposited in the herbarium of the Department of Plant Pathology and Microbiology, National Taiwan University Herbarium (NTUH)^2^. Living cultures are stored at the Department of Plant Pathology and Microbiology, National Taiwan University Culture Collection (NTUCC)^2^. Taxonomic descriptions and nomenclature details were deposited in MycoBank.

### DNA extraction, PCR amplification and sequencing

Total genomic DNA was extracted from axenic isolates grown for 7 d on PDA using the Bioman Fungus Genomic DNA Extraction Kit (BIOMAN) following the manufacturer’s protocol (Bioman Scientific Co., Ltd). PCR amplification was done following the methods described in Ariyawansa et al.^1^ The PCR reactions for amplification of the internal transcribed spacer (ITS) regions^44^, were executed under standard conditions^45,46^. PCR conditions for amplification of the partial SSU (small subunit of the nrRNA gene) and LSU (large subunit of the nrRNA gene) followed the protocol of White et al.^47^. Amplification of partial *tub*2 (β-tubulin), *rpb*2 (partial RNA polymerase II second largest subunit gene) and *tef*1 (partial translation elongation factor 1-α gene) followed the procedures of Woudenberg et al.^30^ and Ariyawansa et al.^1,2^. Primer sets used for these genes are in Table 1.

**Table 1:** Primers used for amplification and sequencing.

Gene region	Primer pairs: sequence (5′–3′)	References
ITS	ITS5: GGAAGTAAAAGTCGTAACAAGG	^44,45^
	ITS4: TCCTCCGCTTATTGATATGC	
LSU	LROR: ACCCGCTGAACTTAAGC	^44,45^
	LR5: TCCTGAGGGAAACTTCG	
SSU	NS1: GTAGTCATATGCTTGTCTC	^44,45^
	NS4: CTTCCGTCAATTCCTTTAAG	
*tub*2	BT2A: GGTAACCAAATCGGTGCTGCTTTC	^47^
	BT2B: ACCCTCAGTGTAGTGACCCTTGGC	
*tef*1	EF1-983F: GCYCCYGGHCAYCGTGAYTTYAT	^48^
	EF1-2218R: GACTTGACTTCRGTVGTGAC	
*rpb*2	RPB2-5F: GAYGAYMGWGATCAYTTYGG	^49^
	RPB2-7cR: CCCATRGCTTGYTTRCCCAT	

Agarose gels (1.5%) stained with SYBR safe DNA gel stain (BIOMAN) were used to visualise the PCR products. PCR products were purified and sequenced by Genomics (New Taipei, Taiwan) using the Sanger sequencing method. SeqMan Pro v.8.1.3 (Lasergene, DNASTAR, Inc., Madison, WI, USA) was used to obtain consensus sequences from sequences generated using forward and reverse primers. Newly obtained sequences were deposited at NCBI GenBank under the accession numbers provided in Supplementary Table S1.

### Sequence alignment and phylogenetic analysis

MAFFT v. 6.864b was used to produce multiple sequence alignments (https://mafft.cbrc.jp/alignment/server/index.html). MEGA v. 5^50^ was used to check alignments visually and adjust manually where required. All introns and exons were aligned individually. Previously published sequences^1–5,10,15,18,19^ were obtained from GenBank and are listed in Supplementary Table S1. Single alignments for each locus and the combined gene datasets were analysed using different tree inference methods. Two different datasets were organized to infer two phylogenies. The first tree focused on phylogenetic placement of the new genus and species introduced in this study in the Pleosporales. The second tree was used to determine the evolutionary placement of *Didymosphaeria* and allied taxa within the family Didymosphaeriaceae.

MrModeltest v. 2.3^51^ with the Akaike Information Criterion (AIC) implemented in PAUP v. 4.0b10 was used to determine evolutionary models for each locus individually. RAxML-HPC2 on XSEDE (v 8.2.8)^52^ with default parameters and bootstrapping with 1,000 replicates was used to conduct maximum likelihood (ML) analysis and was executed in the CIPRES webportal^53^. Another analysis of the same dataset was performed using Bayesian Inference (BI) as implemented in MrBayes ver. 3.1.2^54^. The number of generations was set at 10 million and the run was stopped automatically when the average standard deviation of split frequencies fell below 0.01. Trees were saved every 1,000 generations. The MCMC heated chain was set with a “temperature” value of 0.15. The distribution of log-likelihood scores was checked with Tracer v 1.5 to determine the stationary phase for each search and to decide if extra runs were required to achieve convergence^10,55^. All sampled topologies below the asymptote (20%) were discarded as part of a burn-in procedure, and the remaining trees were used to calculate posterior probabilities in the majority rule consensus tree.

For the concatenated gene analyses the topologies of the trees inferred for single genes were evaluated visually to confirm that the overall tree topology of the single locus datasets were comparable to each other and to that of the tree acquired from the concatenated datas*et al*ignment. ML bootstrap values (MLBS) equal to or greater than 70% and Bayesian posterior probability (PP) equal to or greater than 0.95 are given at each node (Figs. 1, 2). Nodes with bootstrap support (BS) lower than 70% or PP lower than 0.95 were considered unresolved. Phylogenetic trees and data files were viewed in MEGA v. 5^50^ and FigTree v. 1.4^56^.

## Supplementary information

Supplementary Information 1.

Supplementary Information 2.

Supplementary Information 3.
