# Supplementary material for: Species diversity of Pleosporalean taxa associated with Camellia sinensis (L.) Kuntze in Taiwan
Source: Sci Rep. 2020 Jul 29;10:12762. doi: 10.1038/s41598-020-69718-0 (PMC7391694; doi:10.1038/s41598-020-69718-0)
Supplement: Supplementary file 1 — Supplementary Information 1. [file 41598_2020_69718_MOESM1_ESM.docx]

**Species diversity of Pleosporalean taxa associated with *Camellia sinensis* (L.) Kuntze in Taiwan**

**Hiran A. Ariyawansa^1,*^, Ichen Tsai^1^, Kasun M. Thambugala^2^, Wei-Yu Chuang^1^, Shiou-Ruei Lin^3^, Wael N. Hozzein^4,5,^ Ratchadawan Cheewangkoon^6,7,*^**

^1^Department of Plant Pathology and Microbiology, National Taiwan University, College of Bio-Resources and Agriculture, Taipei City, 10617, Taiwan

^2^Genetics and Molecular Biology Unit, Faculty of Applied Sciences, University of Sri Jayewardenepura, Gangodawila, Nugegoda, Sri Lanka.

^3^Department of Tea Agronomy, Tea Research and Extension Station, Taoyuan City, 32654, Taiwan

^4^Bioproducts Research Chair, Zoology Department, College of Science, King Saud University, Riyadh, 11451, Saudi Arabia

^5^Botany and Microbiology Department, Faculty of Science, Beni-Suef University, Beni-Suef, 62521, Egypt

^6^Department of Entomology and Plant Pathology, Faculty of Agriculture, Chiang Mai University, Chiang Mai, 50200, Thailand

^7^Innovative Agriculture Research Centre, Faculty of Agriculture, Chiang Mai University, Chiang Mai, 50200, Thailand

*Corresponding authors: [ariyawansa44@ntu.edu.tw](mailto:ariyawansa44@ntu.edu.tw); ratchadawan.c@cmu.ac.th

**Supplementary data S1. Single gene phylogenies of Pleosporales based on six molecular markers (ITS, LSU, SSU, *rpb2*, *tef1* and *tub2*)**


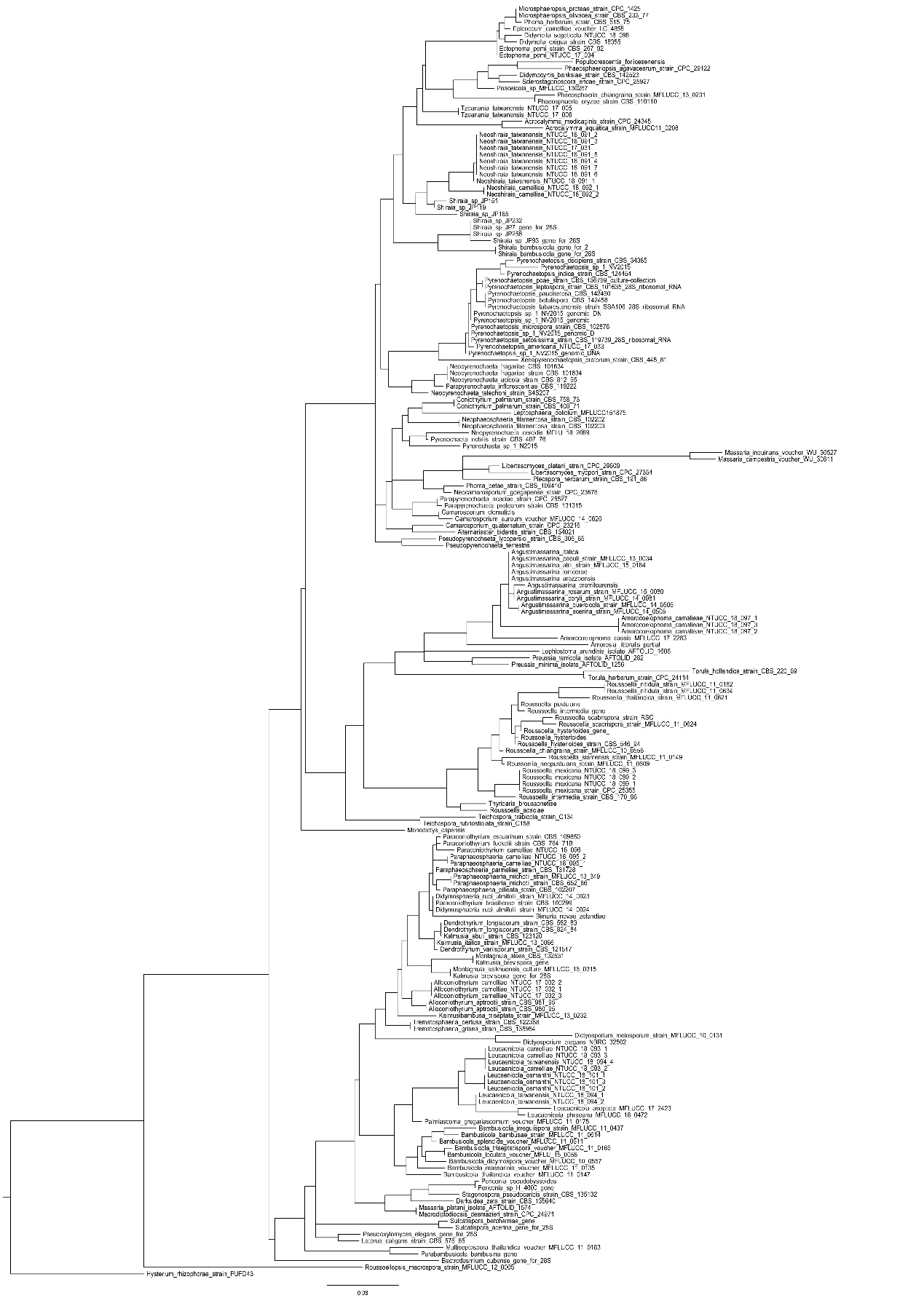


Figure S1. Phylogenetic tree based on the alignment of ITS evaluated using RAxML.


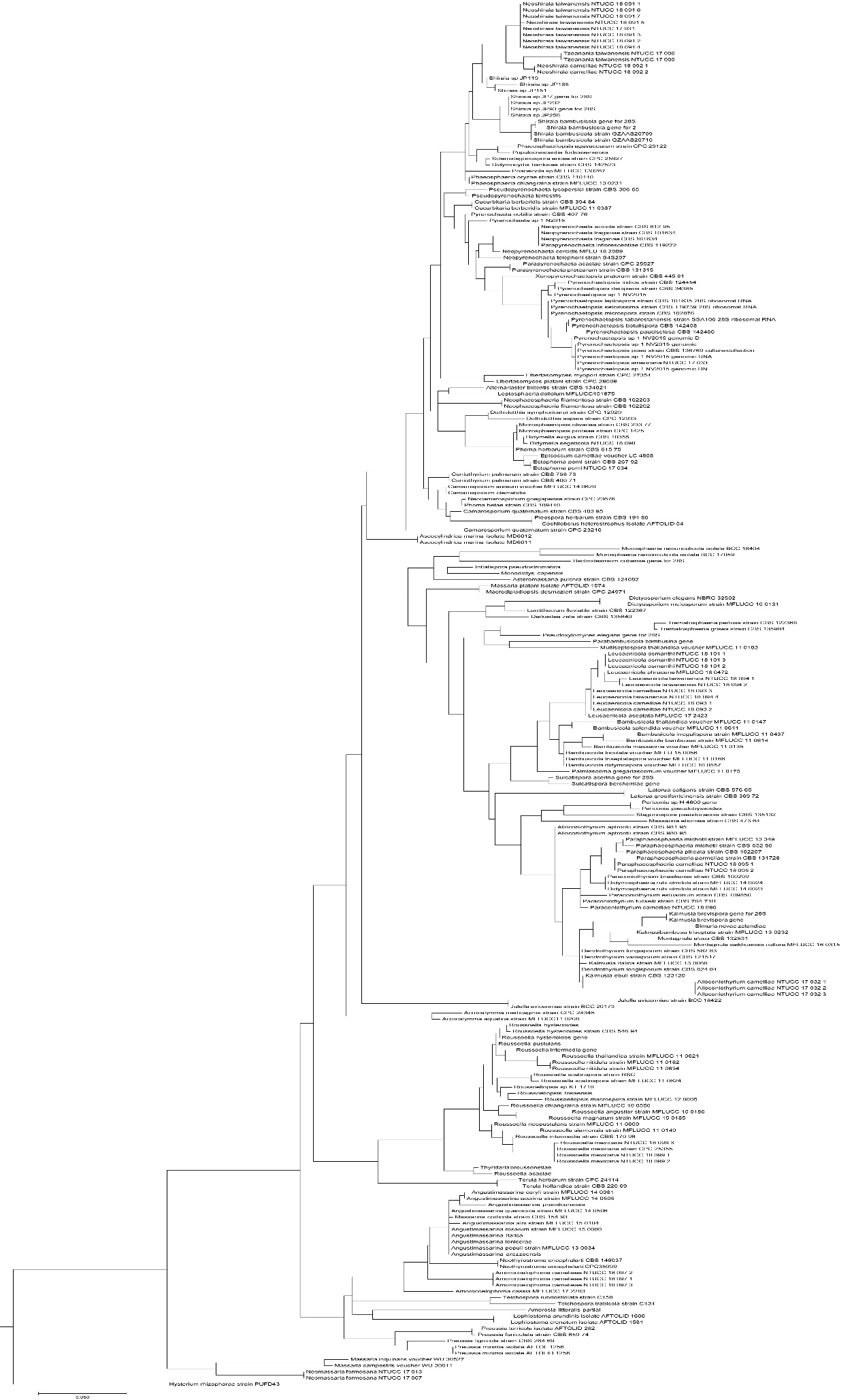


Figure S2. Phylogenetic tree based on the alignment of LSU evaluated using RAxML.


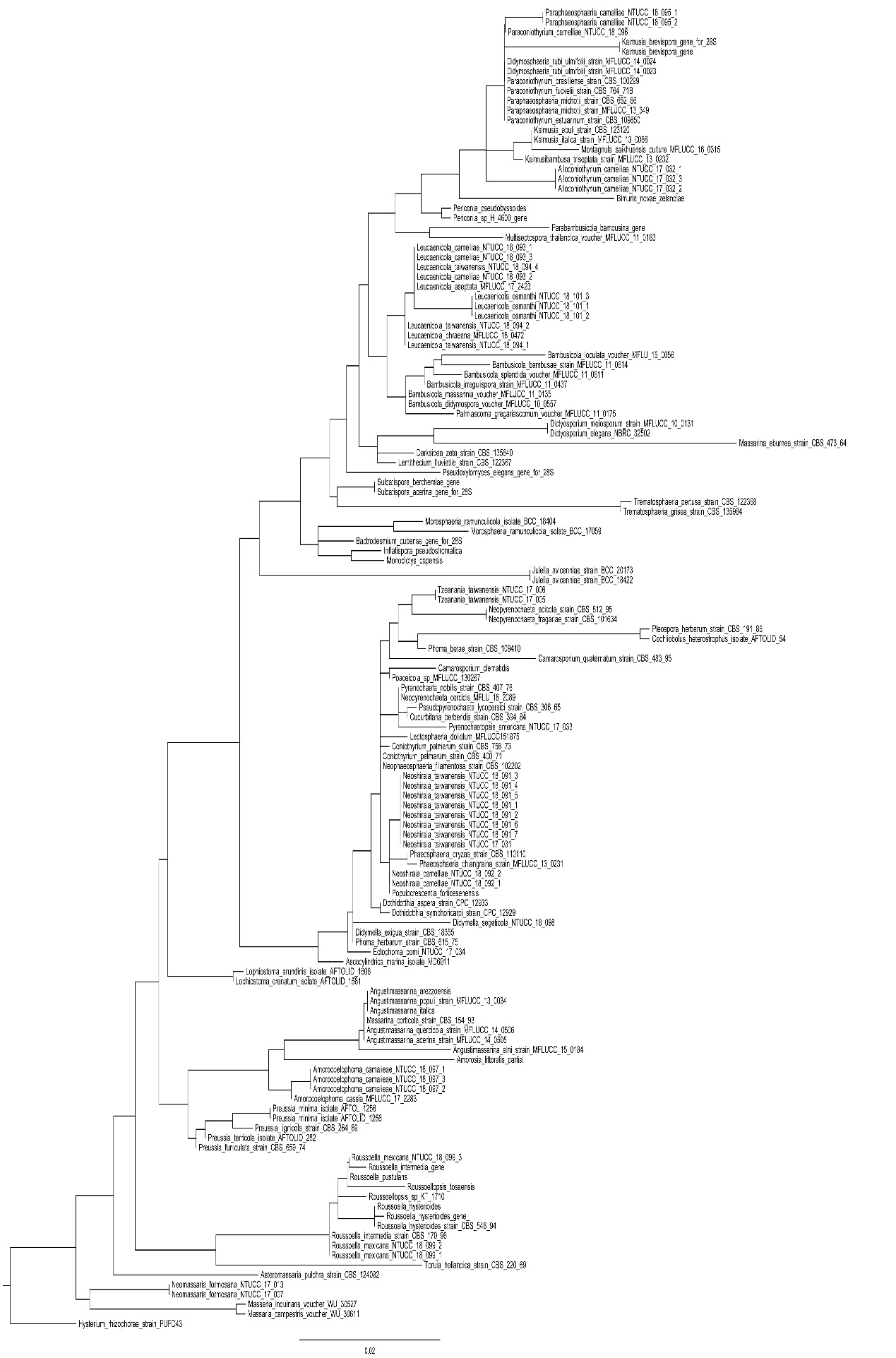


Figure S3. Phylogenetic tree based on the alignment of SSU evaluated using RAxML.


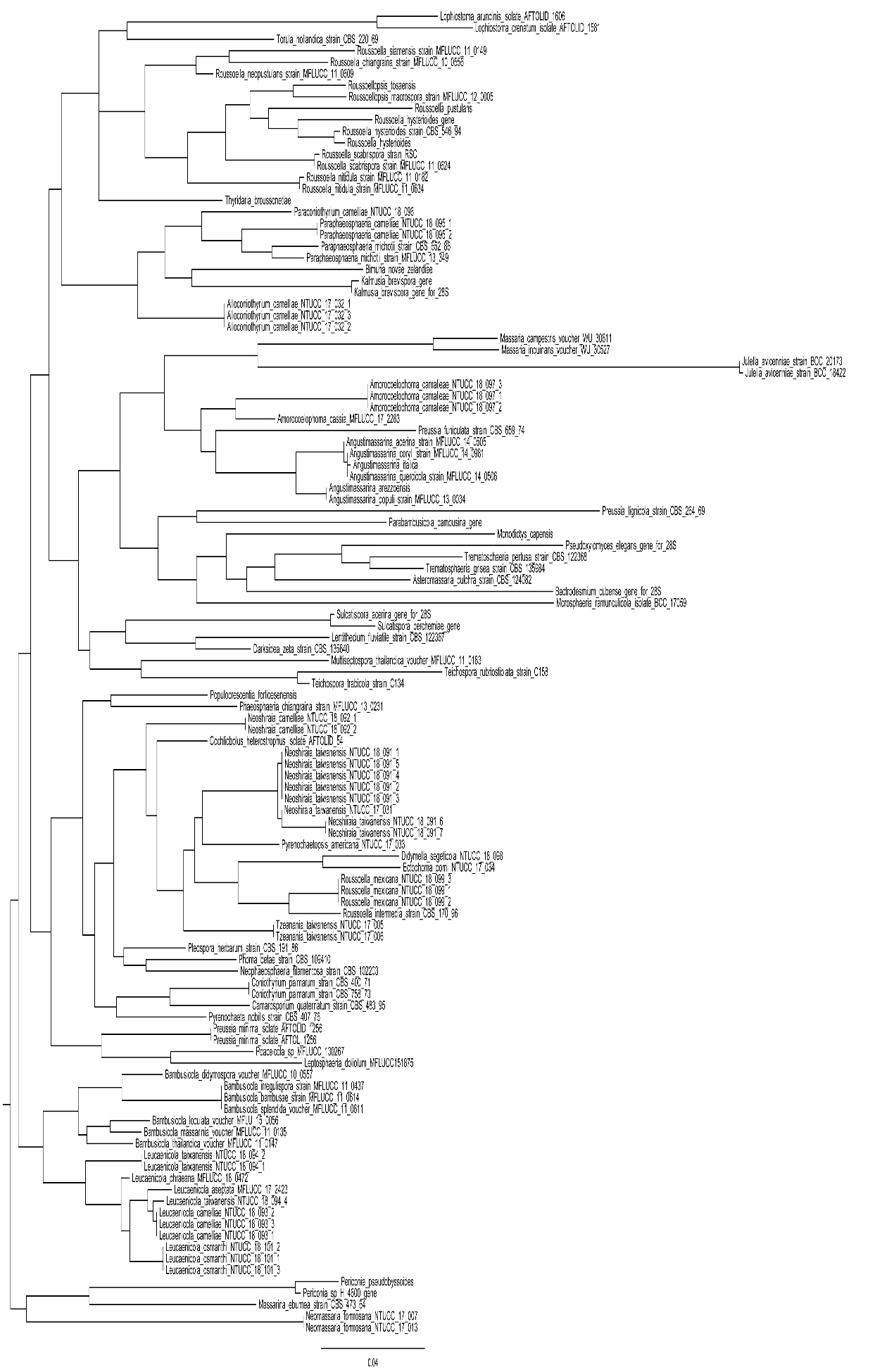


Figure S4. Phylogenetic tree based on the alignment of *tef1* evaluated using RAxML.


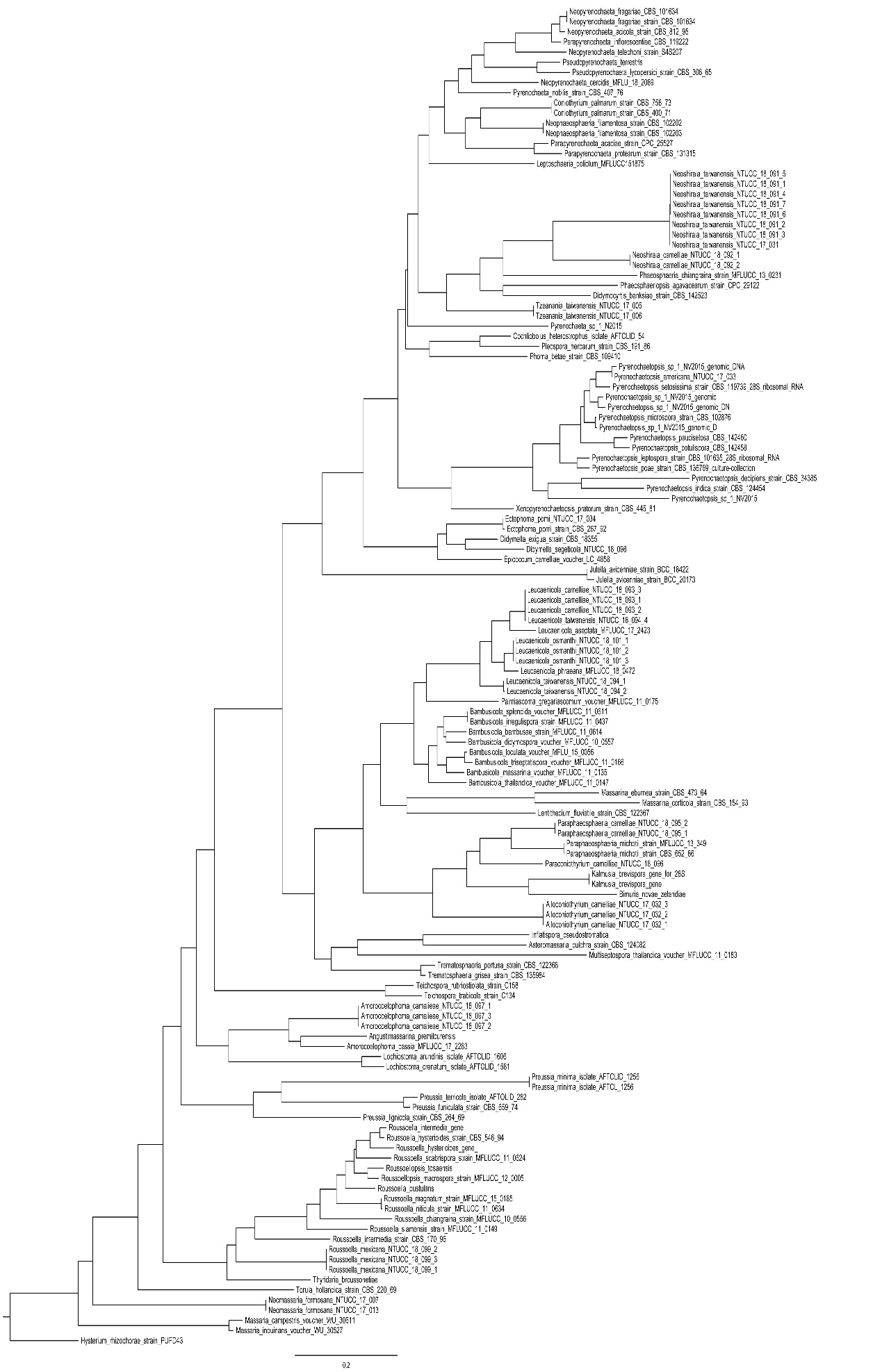


Figure S5. Phylogenetic tree based on the alignment of *rpb2* evaluated using RAxML.


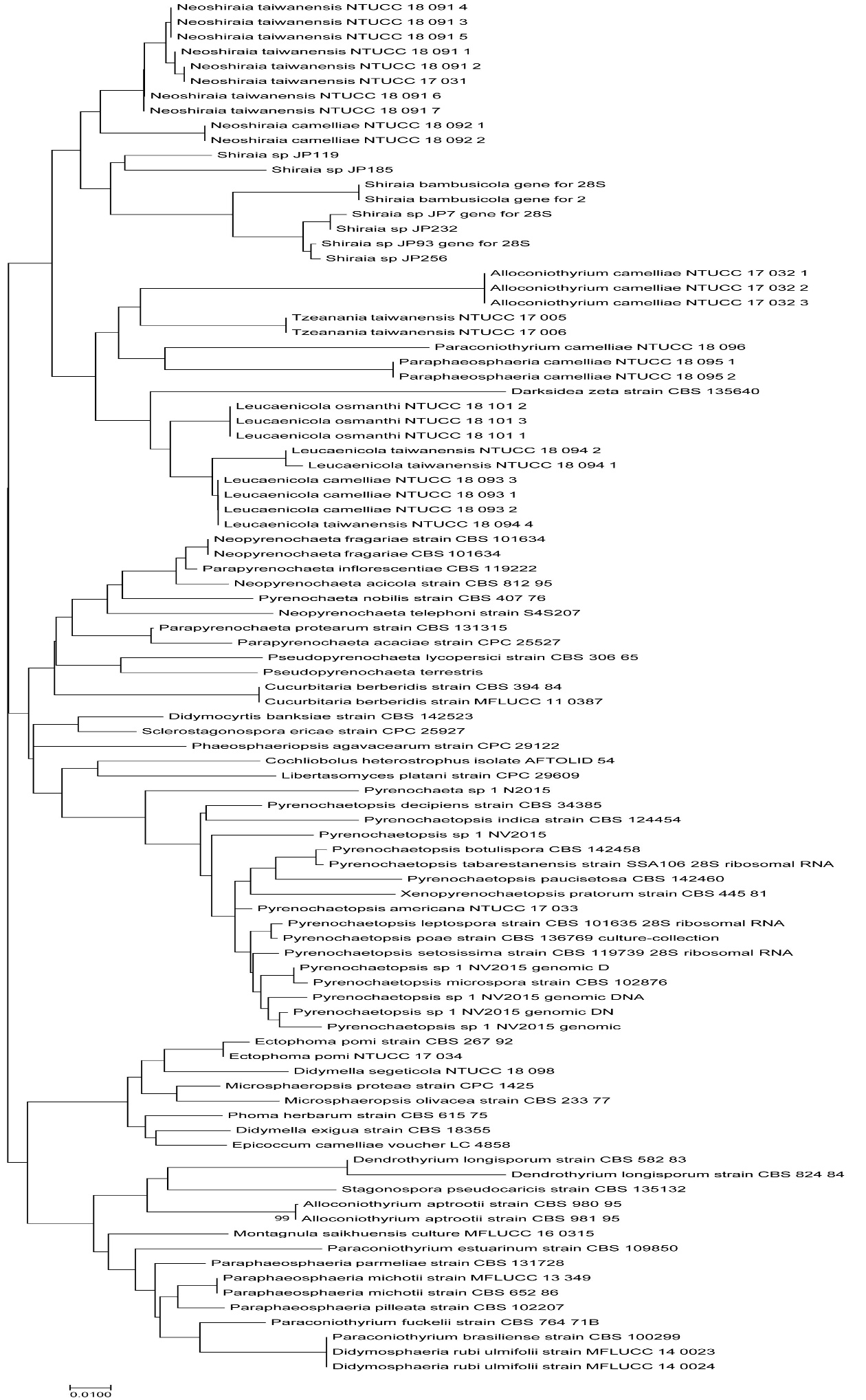


Figure S6. Phylogenetic tree based on the alignment of *tub2* evaluated using RAxML.

**Supplementary data S2. Single gene phylogenies of Didymosphaeriaceae based on four molecular markers (ITS, LSU, SSU, and *tub2*)**

**
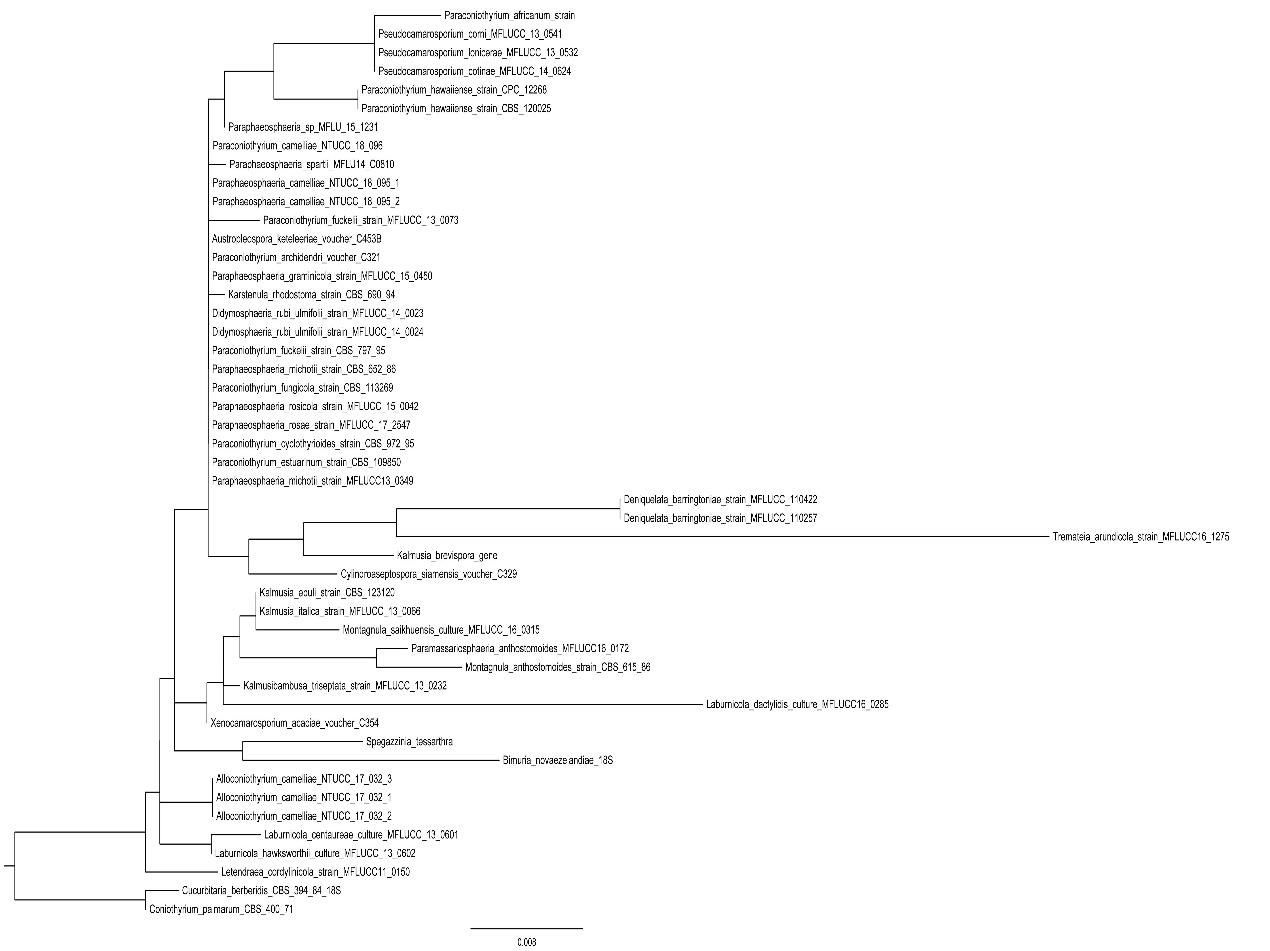
**

Figure S1. Phylogenetic tree based on the alignment of SSU evaluated using RAxML.


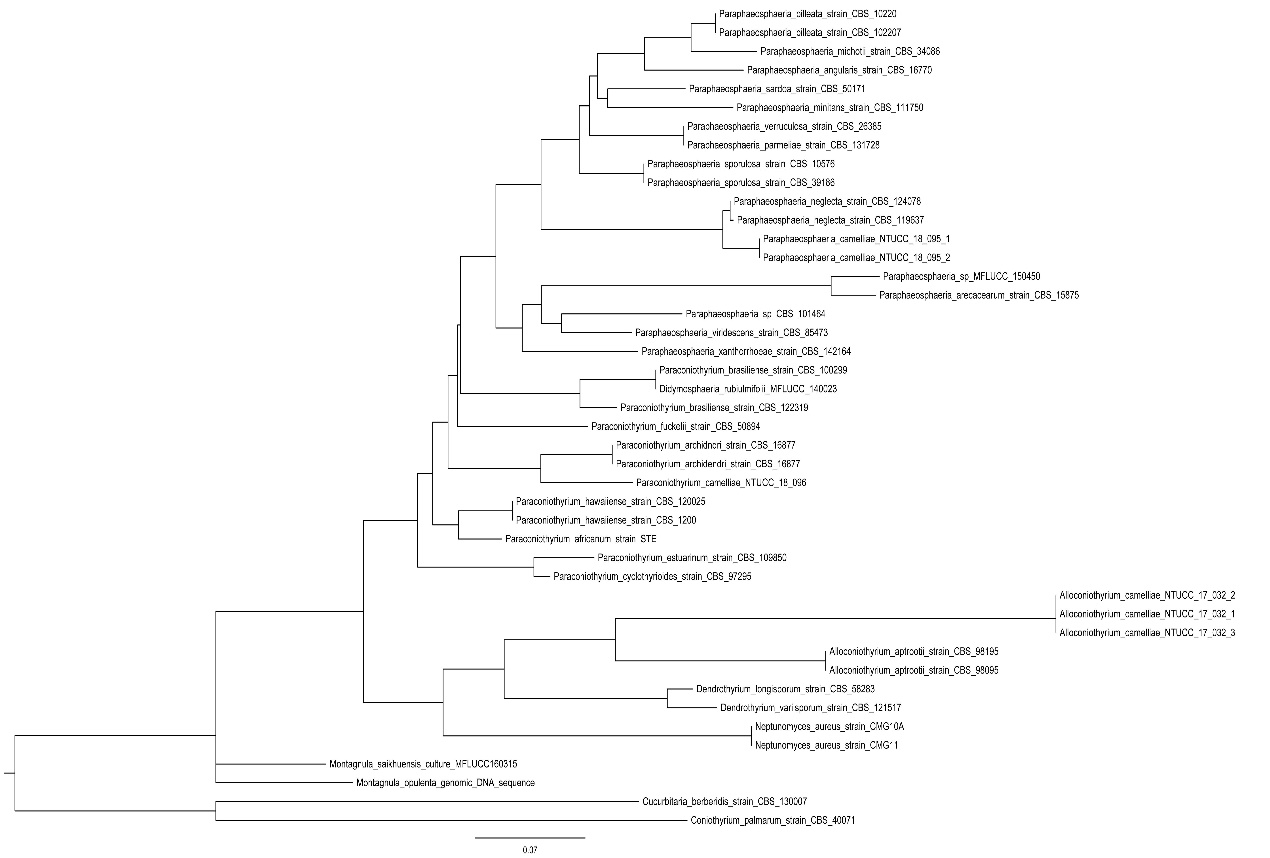


Figure S2. Phylogenetic tree based on the alignment of *tub2* evaluated using RAxML.


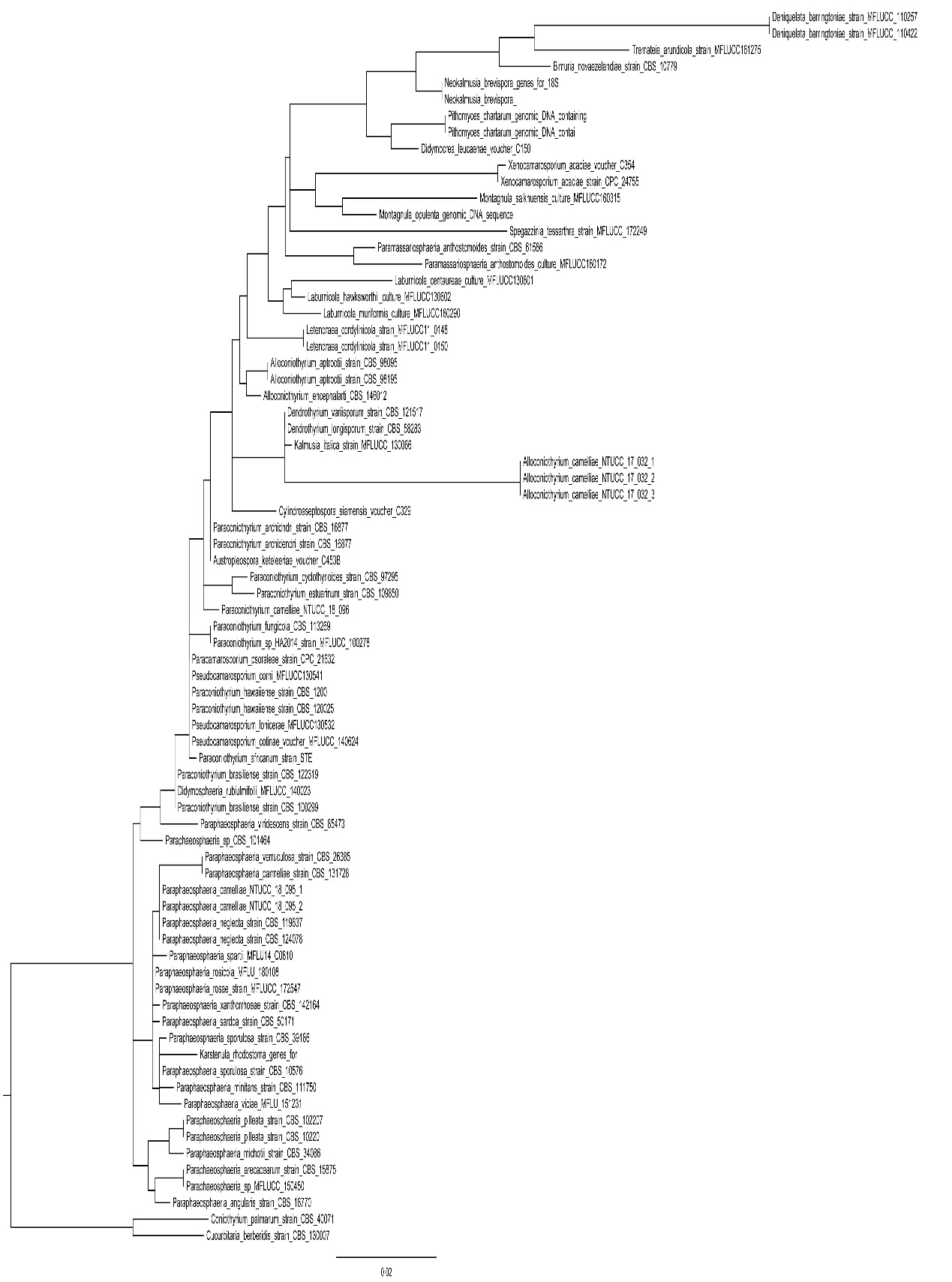


Figure S3. Phylogenetic tree based on the alignment of LSU evaluated using RAxML.


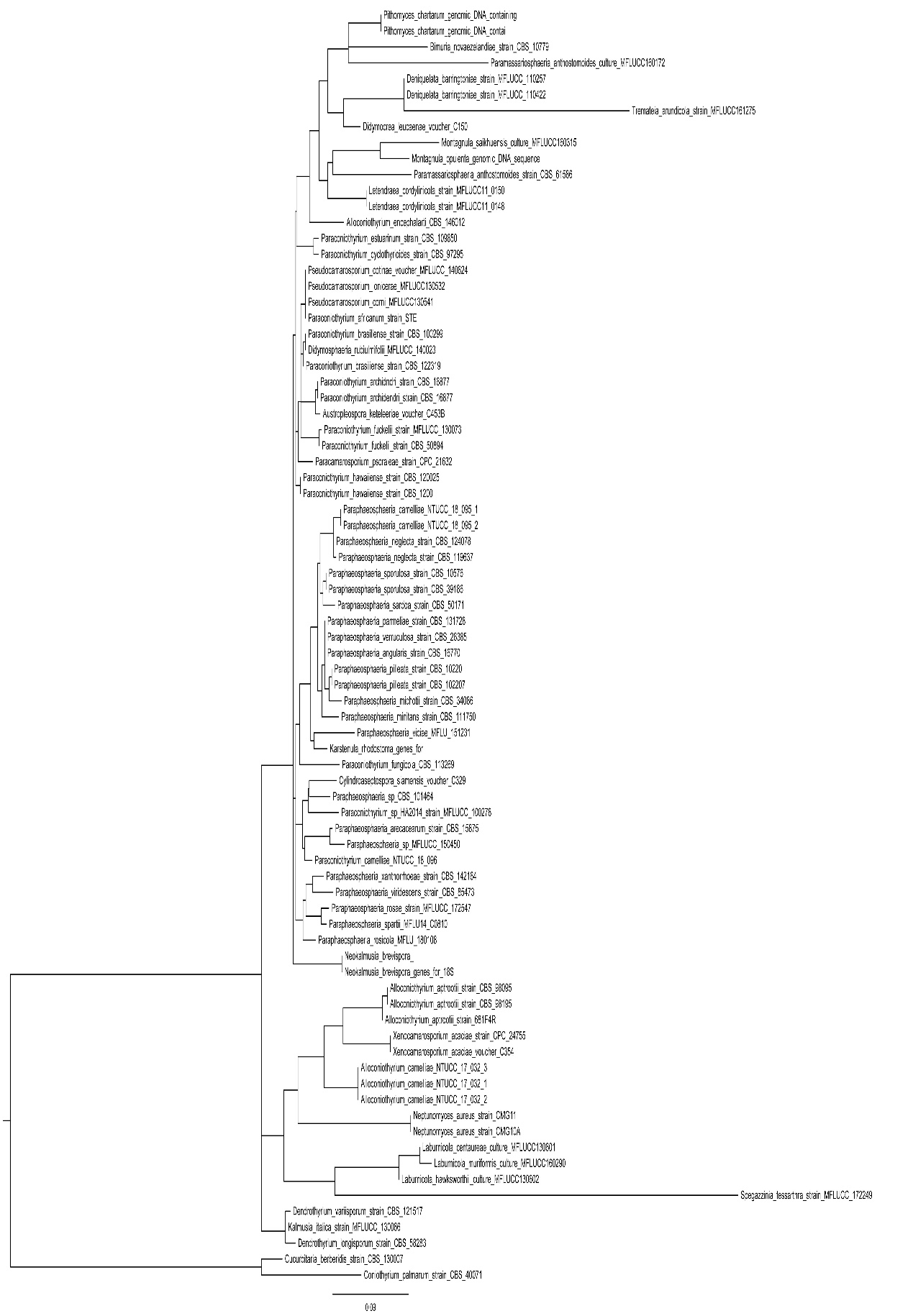


Figure S3. Phylogenetic tree based on the alignment of ITS evaluated using RAxML.
