# Supplementary material for: Species diversity of Pleosporalean taxa associated with Camellia sinensis (L.) Kuntze in Taiwan
Source: Sci Rep. 2020 Jul 29;10:12762. doi: 10.1038/s41598-020-69718-0 (PMC7391694; doi:10.1038/s41598-020-69718-0)
Supplement: Supplementary file 2 — Supplementary Information 2. [file 41598_2020_69718_MOESM2_ESM.docx]

**Species diversity of Pleosporalean taxa associated with *Camellia sinensis* (L.) Kuntze in Taiwan**

**Hiran A. Ariyawansa^1,*^, Ichen Tsai^1^, Kasun M. Thambugala^2^, Wei-Yu Chuang^1^, Shiou-Ruei Lin^3^, Wael N. Hozzein^4,5,^ Ratchadawan Cheewangkoon^6,7,*^**

^1^Department of Plant Pathology and Microbiology, National Taiwan University, College of Bio-Resources and Agriculture, Taipei City, 10617, Taiwan

^2^Genetics and Molecular Biology Unit, Faculty of Applied Sciences, University of Sri Jayewardenepura, Gangodawila, Nugegoda, Sri Lanka.

^3^Department of Tea Agronomy, Tea Research and Extension Station, Taoyuan City, 32654, Taiwan

^4^Bioproducts Research Chair, Zoology Department, College of Science, King Saud University, Riyadh, 11451, Saudi Arabia

^5^Botany and Microbiology Department, Faculty of Science, Beni-Suef University, Beni-Suef, 62521, Egypt

^6^Department of Entomology and Plant Pathology, Faculty of Agriculture, Chiang Mai University, Chiang Mai, 50200, Thailand

^7^Innovative Agriculture Research Centre, Faculty of Agriculture, Chiang Mai University, Chiang Mai, 50200, Thailand

*Corresponding authors: [ariyawansa44@ntu.edu.tw](mailto:ariyawansa44@ntu.edu.tw); ratchadawan.c@cmu.ac.th

Supplementary Table S1. GenBank accession numbers of isolates used for phylogenetic reconstruction. Newly generated isolates are in red

| Taxon | Culture code | LSU | ITS | SSU | *rpb2* | *tef1* | *tub2* |
| --- | --- | --- | --- | --- | --- | --- | --- |
| *Leptosphaeria doliolum* | MFLUCC 15-1875 | KT454719 | KT454727 | KT454734 | KY064035 | GU349069 | JF740144 |
| *Alternariaster bidentis* | CBS 134021 | KC609341 | KC609333 |  |  |  |  |
| *Bipolaris maydis* | CBS 134.39 | AY544645 |  | AY544727 | DQ247790 | DQ497603 | XM_014226937 |
| *Pleospora herbarum* | CBS 191.86 | DQ247804 | KC584239 | GU238232 | DQ247794 | DQ471090 | AY749032 |
| *Coniothyrium palmarum* | CBS 758.73 | EU754153 | AY720708 | AY642513 | DQ677956 | DQ677903 |  |
| *Coniothyrium palmarum* | CBS 400.71 | JX681085 |  | EU754055 |  |  |  |
| *Dothidotthia aspera* | CPC 12933 | EU673276 |  | EU673228 |  |  |  |
| *Dothidotthia symphoricarpi* | CPC 12929 | EU673273 |  | EU673224 |  |  |  |
| *Neophaeosphaeria filamentosa* | CBS 102203 | GQ387577 | JF740259 | GQ387516 |  |  |  |
| *Neophaeosphaeria filamentosa* | CBS 102202 | JX681104 |  |  | GU371773 | GU349084 |  |
| *Halojulella avicenniae* | BCC 20173 | GU371822 |  | GU371830 | GU371786 | GU371815 |  |
| *Halojulella avicenniae* | BCC 18422 | GU371823 |  |  | GU371787 | GU371816 |  |
| *Microsphaeropsis proteae* | CPC 1425 | JN712563 | JN712497 |  |  |  | JN712650 |
| *Microsphaeropsis olivacea* | CBS 233.77 | GU237988 | GU237803 |  |  |  | GU237549 |
| *Libertasomyces myopori* | CPC 27354 | KX228332 | KX228281 |  |  |  |  |
| *Libertasomyces platani* | CPC 29609 | KY173507 | KY173416 |  | KY173585 |  | KY173604 |
| *Neocamarosporium goegapense* | CBS 138008 | KJ869220 | KJ869163 |  |  |  |  |
| *Neocamarosporium betae* | CBS 109410 | EU754178 | KY940790 | EU754079 | GU371774 | GU349075 |  |
| *Phaeosphaeria chiangraina* | MFLUCC 13-0231 | KM434280 | KM434270 | KM434289 | KM434307 | KM434298 |  |
| *Phaeosphaeria oryzae* | CBS 110110 | KF251689 | KF251186 | GQ387530 |  |  |  |
| *Shiraia bambusicola* | GZAAS2.0710 | KC460984 |  |  |  |  |  |
| *Shiraia bambusicola* | GZAAS2.0709 | KC460983 |  |  |  |  | AB355005 |
| *Shiraia bambusicola* | NBRC 30772 | AB354972 | AB354991 |  |  |  | AB355006 |
| *Shiraia bambusicola* | NBRC 30771 | AB354971 | AB354990 |  |  |  |  |
| *Shiraia sp.* | JP7 | AB354974 | AB255241 |  |  |  | AB355008 |
| *Shiraia sp.* | JP93 | AB354975 | AB255277 |  |  |  | AB355009 |
| *Shiraia sp.* | JP119 | AB354976 | AB354993 |  |  |  | AB355010 |
| *Shiraia sp.* | JP151 | AB354977 | AB255289 |  |  |  |  |
| *Shiraia sp.* | JP185 | AB354978 | AB354994 |  |  |  | AB355011 |
| *Shiraia sp.* | JP232 | AB354979 | AB255303 |  |  |  | AB355012 |
| *Shiraia sp.* | JP256 | AB354980 | AB354995 |  |  |  | AB355013 |
| *Didymella segeticola* | CGMCC 3.17498 | KP330454 | KP330442 |  | KP330413 |  | KP330398 |
| *Ectophoma pomi* | CBS 267.92T | GU238128 | GU237814 |  | LT623263 |  | GU237643 |
| *Phoma herbarum* | CBS 615.75 | KF251715 | FJ427022 | EU754087 | KF252217 | DQ677909 | KF252703 |
| *Epicoccum camelliae* | CGMCC 3.18343T | KY742245 | KY742091 |  | KY742170 |  | KY742333 |
| *Cucurbitaria berberidis* | CBS 394.84 | JX681088 |  | GQ387544 |  |  | KT389794 |
| *Cucurbitaria berberidis* | MFLUCC 11-0387 | KC506796 |  | KC506800 |  |  |  |
| *Pyrenochaeta_nobilis_* | CBS_407_76 | EU754206 | EU930011 | EU754107 | LT623276 | MF795880 | KT389845 |
| *Pyrenochaetopsis botulispora* | CBS 142458 | LN907441 | LT592946 |  | LT593080 |  | LT593010 |
| *Pyrenochaetopsis paucisetosa* | CBS 142460 | LN907336 | LT592897 |  | LT593035 |  | LT592966 |
| *Xenopyrenochaetopsis pratorum* | CBS 445.81T | GU238136 | JF740263 |  | KT389671 |  | KT389846 |
| *Parapyrenochaeta protearum* | CBS 131315 | JQ044453 | JQ044434 |  | LT717683 |  | LT717677 |
| *Parapyrenochaeta acaciae* | CBS 141291 | KX228316 | KX228265 |  | LT717686 |  | LT717679 |
| *Neopyrenochaeta fragariae* | CBS 101634 | GQ387603 | LT623217 | GQ387542 | LT623270 |  | LT623231 |
| *Neopyrenochaeta acicola* | CBS 812.95 | GQ387602 | LT623218 | GQ387541 | LT623271 |  | LT623232 |
| *Neopyrenochaeta cercidis* | MFLU 18-2089 | MK347932 | MK347718 | MK347823 | MK434908 |  |  |
| *Neopyrenochaeta inflorescentiae* | CBS 119222T | EU552153 | EU552153 |  | LT623272 |  | LT623233 |
| *Neopyrenochaeta telephoni* | CBS 139022T | KM516290 | KM516291 |  | LT717685 |  | LT717678 |
| *Pseudopyrenochaeta lycopersici* | CBS 306.65 | EU754205 | NR_103581 | EU754106 | LT717680 |  | LT717674 |
| *Pseudopyrenochaeta terrestris* | CBS 282.72 | LT623216 | LT623228 |  | LT623287 |  | LT623246 |
| *Camarosporidiella clematidis* | MFLUCC 12-0354 | KJ562188 | KJ562213 | KJ589414 |  |  |  |
| *Camarosporidiella aureum* | MFLUCC 14-0620 | KP744478 | KP744436 | KP753948 |  |  |  |
| *Camarosporium quaternatum* | CBS 483.95 | GU301806 |  | GU296141 |  | GU349044 |  |
| *Alloconiothyrium aptrootii* | CBS 980.95 | JX496234 | JX496121 |  |  |  | JX496460 |
| *Alloconiothyrium aptrootii* | CBS 981.95 | JX496235 | JX496122 |  |  |  | JX496461 |
| *Bimuria novae-zelandiae* | CBS 107.79 | AY016356 | MG813173 | AY016338 | DQ470917 | DQ471087 |  |
| *Didymosphaeria rubi-ulmifolii* | CBS 100299 | JX496124 | AY642531 |  |  |  | JX496350 |
| *Didymosphaeria rubi-ulmifolii* | MFLUCC 140023 | KJ436586 | KJ436586 | KJ436588 |  |  | KJ939276 |
| *Didymosphaeria rubi-ulmifolii* | MFLUCC 140024 | KJ436585 | KJ436585 | KJ436587 |  |  | KJ939277 |
| *Kalmusia ebuli* | CBS 123120 | JN644073 | KF796674 | JN851818 |  |  |  |
| *Kalmusia longisporum* | CBS 582.83 | JX496210 | JX496097 |  |  |  | JX496436 |
| *Kalmusia longisporum* | CBS 824.84 | JX496228 | JX496097 |  |  |  | JX496454 |
| *Kalmusibambusa triseptata* | MFLUCC 13-0232 | KY682695 | KY682697 | KY682696 |  |  |  |
| *Kalmusia italica* | MFLUCC 13-0066 | KP325441 | KP325440 | KP325442 |  |  |  |
| *Kalmusia variisporum* | CBS 121517 | JX496143 | JX496030 |  |  |  | JX496369 |
| *Montagnula saikhuensis* | MFLUCC:16-0315 | KU743210 | KU743209 | KU743211 |  |  | KU743216 |
| *Montagnula aloes* | CPC 19671 | JX069847 | NR_111757 |  |  |  |  |
| *Neokalmusia brevispora* | KT 1466 | AB524600 | KU743209 | AB524459 | AB539099 | AB539112 |  |
| *Neokalmusia brevispora* | KT 2313 | AB524601 | JX069863 | AB524460 | AB539100 | AB539113 |  |
| *Paraconiothyrium estuarinum* | CBS 109850 | JX496129 | JX496016 | AY642522 |  |  | JX496355 |
| *Paraconiothyrium fuckelii* | CBS 764.71B | JX496225 | JX496112 | GU238204 |  |  | JX496451 |
| *Paraphaeosphaeria michotii* | MFLUCC 130349 | KJ939282 | KJ939279 | KJ939285 |  | AB808535 |  |
| *Paraphaeosphaeria michotii* | CBS 652.86 | JX496216 | JX496103 | GQ387520 | GU456351 | GU456266 | JX496442 |
| *Paraphaeosphaeria pilleata* | CBS 102207 | JX496126 | JX496013 |  |  |  | JX496352 |
| *Paraphaeosphaeria parmeliae* | CBS 131728 | KP170722 | KP170654 |  |  | KP170679 | KP170703 |
| *Massarina eburnea* | CBS 473.64 | GU301840 |  | GU296170 | GU371732 | GU349040 |  |
| *Stagonospora pseudocaricis* | CBS 135132 HT | KF251762 | KF251259 |  | KF252264 | KF253209 | KF252741 |
| *Periconia pseudobyssoides* | H 4151 | AB807568 | LC014587 | AB797278 |  | AB808544 |  |
| *Periconia byssoides* | H 4432 | AB807570 | LC014581 | AB797280 |  | AB808546 |  |
| *Dictyosporium elegans* | NBRC 32502 | DQ018100 | DQ018087 | DQ018079 |  |  |  |
| *Dictyosporium meiosporum* | MFLUCC 10-0131 | KP710945 | KP710944 | KP710946 |  |  |  |
| *Lentithecium fluviatile* | CBS 122367 | FJ795451 |  | GU296158 | FJ795467 | GU349074 |  |
| *Darksidea zeta* | CBS 135640 HT | KP184013 | KP183979 | KP184071 |  | KP184191 | KP184204 |
| *Latorua caligans* | CBS 576.65 HT | KR873266 | KR873232 |  |  |  |  |
| *Latorua grootfonteinensis* | CBS 369.72 HT | KR873267 |  |  |  |  |  |
| *Sulcatispora berchemiae* | KT 1607 HT | AB807534 | AB809635 | AB797244 |  | AB808509 |  |
| *Sulcatispora acerina* | KT 2982 HT | LC014610 | LC014597 | LC014605 |  | LC014615 |  |
| *Parabambusicola bambusina* | H 4321 | AB807536 | LC014578 | AB797246 |  | AB808511 |  |
| *Multiseptospora thailandica* | MFLUCC 11–0183 | KP744490 | KP744447 | KP753955 | KU705662 | KU705657 |  |
| *Macrodiplodiopsis desmazieri* | CBS 140062 ET | KR873272 | NG_058182 |  |  |  |  |
| *Macrodiplodiopsis desmazieri* | CBS 221.37 | DQ678065 | NR_132924 |  |  |  |  |
| *Pseudoxylomyces elegans* | KT 2887 | AB807598 | LC014593 | AB797308 |  | AB808576 |  |
| *Trematosphaeria pertusa* | CBS 122368 ET | FJ201990 | KF015668 | FJ201991 | FJ795476 | KF015701 |  |
| *Trematosphaeria grisea* | CBS 332.50 HT | KF015618 | KF015666 | KF015632 | KF015724 | KF015694 |  |
| *Bactrodesmium cubense* | CBS 680.96 | AB807508 | LC014541 | AB797218 |  | AB808484 |  |
| *Morosphaeria ramunculicola* | BCC 18404 | GQ925853 |  | GQ925838 |  |  |  |
| *Morosphaeria velatispora* | KH 218 | GQ925852 |  | GQ925841 |  | AB808532 |  |
| *Inflatispora pseudostromatica* | IFRD 2013 | JN231131 |  | JN231132 | JN231133 |  |  |
| *Monodictys capensis* | HR 1 | AB807551 | LC014570 | AB797261 |  | AB808527 |  |
| *Asteromassaria pulchra* | CBS 124082 | GU301800 |  | GU296137 | GU371772 | GU349066 |  |
| *Bambusicola splendida* | MFLUCC 11-0439 | KU863110 | NR_121549 | JX442042 | KU940168 | KP761726 |  |
| *Bambusicola irregulispora* | MFLUCC 11-0437 | JX442036 | NR_121547 | JX442040 | KP761719 | KP761723 |  |
| *Bambusicola bambusae* | MFLUCC 11-614 | JX442035 | NR_121546 | JX442039 | KP761718 | KP761722 |  |
| *Bambusicola didymospora* | MFLUCC 10–0557 | KU863105 | KU940117 | KU872111 | KU940164 | KU940188 |  |
| *Bambusicola loculata* | MFLUCC 13-0856 | KP761729 | KP761732 | KP761735 | KP761715 | KP761724 |  |
| *Bambusicola triseptatispora* | MFLUCC 11-0166 | KU863109 | NR_153624 |  | KU940167 |  |  |
| *Bambusicola massarinia* | MFLUCC 11-0389 | KU863111 | NR_121548 | KU872115 | KU940169 | KU940192 |  |
| *Bambusicola thailandica* | MFLUCC 11-0147 | KU863108 | KU940119 | KU872113 | KU940166 | KU940191 |  |
| *Palmiascoma gregariascomum* | MFLUCC 11-0175 | KP744495 | NR_154316 | KP753958 | KP998466 |  |  |
| *Acrocalymma medicaginis* | CPC 24340 | KP170718 | KP170625 |  |  |  |  |
| *Acrocalymma aquatica* | MFLUCC11-0208 | JX276952 | NR_121544 | JX276953 |  |  |  |
| *Ascocylindrica marina* | MD6012 | KT252905 |  | KT252907 |  |  |  |
| *Ascocylindrica marina* | MD6011 | KT252906 |  |  |  |  |  |
| *Preussia minima* | CBS 524.50 | DQ678056 | KT389543 | DQ678003 | DQ677950 | DQ677897 |  |
| *Preussia terricola* | DAOM 230091 | AY544686 | KT225529 | AY544726 | DQ470895 | DQ471063 |  |
| *Hysterium rhizophorae* | PUFD43 | MG844276 | MG844284 | MG844280 | MG968956 |  |  |
| *Massaria inquinans* | WU 30527 | HQ599402 | HQ599402 | HQ599402 | HQ599460 | HQ599342 |  |
| *Massaria campestris* | WU 30611 | HQ599385 | HQ599385 | HQ599449 | HQ599459 | HQ599325 |  |
| *Roussoella angustior* | MFLUCC 15-0186 | KT281979 |  |  |  |  |  |
| *Roussoella chiangraina* | MFLUCC 10-0556 | KJ474840 | KJ474828 |  | KJ474857 | KJ474849 |  |
| *Roussoella hysterioides* | CBS 546.94 | KF443381 | KF443405 | AY642528 | KF443392 | KF443399 |  |
| *Roussoella intermedia* | NBRC 106245 | AB524624 | KJ474831 | AB524483 |  |  |  |
| *Roussoella japanensis* | MAFF 239636 | AB524621 | KJ474829 | AB524480 | AB539101 | AB539114 |  |
| *Roussoella magnatum* | MFLUCC 15-0185 | KT281980 |  |  |  |  |  |
| *Roussoella mexicana* | CPC 25355 | KT950862 | KT950848 |  |  |  |  |
| *Roussoella neopustulans* | MFLUCC 11-0609 | KJ474841 | KJ474833 |  |  | KJ474850 |  |
| *Roussoella nitidula* | MFLUCC 11-0182 | KJ474843 | KJ474835 |  | KJ474859 | KJ474852 |  |
| *Roussoella nitidula* | MFLUCC 11-0634 | KJ474842 | KJ474834 |  | KJ474858 | KJ474851 |  |
| *Roussoella pustulans* | MAFF 239637 | AB524623 | KJ474830 | AB524482 | AB539103 | AB539116 |  |
| *Roussoella scabrispora* | MFLUCC 11-0624 | KJ474844 | KJ474836 |  | KJ474860 | KJ474853 |  |
| *Roussoella scabrispora* | WU:33540 | KX650566 | KX650566 |  |  | KX650537 |  |
| *Roussoella siamensis* | MFLUCC 11-0149 | KJ474845 | KJ474837 |  | KJ474861 | KJ474854 |  |
| *Roussoella sp.* | CBS 170.96 | KF443382 | KF443407 | KF443390 | KF443394 | KF443398 |  |
| *Roussoella thailandica* | MFLUCC 11-0621 | KJ474846 | KJ474838 |  |  |  |  |
| *Roussoella verrucispora* | CBS 125434 | AB524622 | KJ474832 | AB524481 | AB539102 | AB539115 |  |
| *Roussoellopsis macrospora* | MFLUCC 12-0005 | KJ474847 | KJ739604 | KJ739608 | KJ474862 | KJ474855 |  |
| *Roussoellopsis sp.* | NBRC 106246 | AB524626 |  | AB524485 |  |  |  |
| *Roussoellopsis tosaensis* | MAFF 239638 | AB524625 |  | AB524484 | AB539104 | AB539117 |  |
| *Thyridaria acaciae* | CBS 138873 | KP004497 | KP004469 |  |  |  |  |
| *Thyridaria broussonetiae* | CBS 121895 | KX650567 | KX650567 |  | KX650585 | KX650538 |  |
| *Torula herbarum* | CBS 140066 | KR873288 | KR873260 |  |  |  |  |
| *Torula hollandica* | CBS 220.69 | KF443384 | KF443406 | KF443389 | KF443393 | KF443401 |  |
| *Amorosia littoralis* | NN 6654 | AM292055 | AM292047 | AM292056 | NA | NA |  |
| *Lophiostoma arundinis* | CBS 621.86 | DQ782384 | AJ496633 | DQ782383 | DQ782386 | DQ782387 |  |
| *Lophiostoma crenatum* | CBS 629.86 | DQ678069 |  | DQ678017 | DQ677965 | DQ677912 |  |
| *Preussia funiculata* | CBS 659.74 | GU301864 |  | GU296187 | GU371799 | GU349032 |  |
| *Preussia lignicola* | CBS 264.69 | GU301872 |  | GU296197 | GU371765 | GU349027 |  |
| *Preussia minima* | CBS 404.59 | MH869446 | MH857904 |  |  |  |  |
| *Teichospora rubriostiolata* | C158 | KU601587 | KU601587 |  | KU601596 | KU601607 |  |
| *Teichospora trabicola* | C134 | KU601591 | KU601591 |  | KU601600 | KU601601 |  |
| *Angustimassarina acerina* | MFLUCC 14–0505 | KP888637 | KP899132 | KP899123 | NA | KR075168 |  |
| *Angustimassarina populi* | MFLUCC 13–0034 | KP888642 | KP899137 | KP899128 | NA | KR075164 |  |
| *Angustimassarina quercicola* | MFLUCC 14–0506 | KP888638 | KP899133 | KP899124 | NA | KR075169 |  |
| *Angustimassarina rosarum* | MFLUCC 15-0080 | MG828985 | MG828869. |  |  |  |  |
| *Angustimassarina arezzoensis* | MFLUCC 13-0578 | KY496722 | KY496743 | KY501113 |  | KY514392 |  |
| *Angustimassarina lonicerae* | MFLUCC15-0087 | KY496724 | KY496759 |  |  |  |  |
| *Angustimassarina italica* | MFLUCC15-0082 | KY496736 | KY496756 | KY501124 |  | KY514400 |  |
| *Angustimassarina premilcurensis* | MFLUCC 15-0074 | KY496725 | KY496745 |  | KY514404 |  |  |
| *Massarina corticola* | CBS 154.93 | FJ795448 |  | FJ795491 | FJ795465 |  |  |
| *Angustimassarina coryli strain* | MFLUCC 14-0981 | MF167432 | MF167431 |  |  | MF167433 |  |
| *Angustimassarina alni strain* | MFLUCC 15-0184 | KY548097 | KY548099 | KY548098 |  |  |  |
| *Roussoella hysterioides* | CBS 125434 | MH875155 | MH863689 |  |  |  |  |
| *Pyrenochaetopsis tabarestanensis* | CBS 139506 | KF803343 | KF730241 |  |  |  | KX789523 |
| *Pyrenochaetopsis leptospora* | CBS 101635T | GQ387627 | JF740262 |  | LT623282 |  | LT623242 |
| *Pyrenochaetopsis poae* | CBS 136769T | KJ869175 | KJ869117 |  | LT623286 |  | KJ869243 |
| *Pyrenochaetopsis setosissima* | CBS 119739T | GQ387632 | LT623227 |  | LT623285 |  | LT623245 |
| *Pyrenochaetopsis americana* | UTHSC DI16-225T | LN907368 | LT592912 |  | LT593050 |  | LT592981 |
| *Pyrenochaetopsis uberiformis* | CBS 142461T | LN907420 | LT592935 |  | LT593074 |  | LT593004 |
| *Pyrenochaetopsis microspora* | UTHSC DI16-198 | LN907341 | LT592899 |  | LT593037 |  | LT592968 |
| *Pyrenochaetopsis microspora* | CBS 102876T | GQ387631 | LT623226 |  | LT623284 |  | LT623244 |
| *Pyrenochaetopsis globosa* | CBS 143034T | LN907418 | LT592934 |  | LT593072 |  | LT593003 |
| *Pyrenochaetopsis decipiens* | CBS 343.85T | GQ387624 | LT623223 |  | LT623280 |  | LT623240 |
| *Pyrenochaetopsis indica* | CBS 124454T | GQ387626 | LT623224 |  | LT623281 |  | LT623241 |
| *Pyrenochaetopsis confluens* | CBS 142459T | LN907446 | LT592950 |  | LT593089 |  | LT593019 |
| *Neopyrenochaetopsis hominis* | CBS 143033T | LN907381 | LT592923 |  | LT593061 |  | LT592992 |
| *Didymocyrtis banksiae* | strain CBS 142523 | KY979812 | NR_154037 |  | KY979850 | KY979895 | KY979850 |
| *Poaceicola italica* | MFLUCC 13-0267T | KX910094 | KX926421 | KX950409 |  | MG520924 |  |
| *Populocrescntia forlicesesensis* | MFLU 15-0651T | KT306952 | KT306948 | KT306955 |  | MG520925 |  |
| *Phaeosphaeriopsis agavacearum* | CPC 29122 | KY173520 | KY173430 |  | KY173591 |  | KY173610 |
| *Sclerostagonospora ericae* | strain CPC 25927 | KX228319 | KX228268 |  |  | KX228375 | KX228383 |
| *Tzeanania taiwanensis* | NTUCC 17-005 | MH461120 | MH461123 | MH461126 | MH461128 | MH461130 | MH461132 |
| *Tzeanania taiwanensis* | NTUCC 17-006 | MH461121 | MH461124 | MH461127 | MH461129 | MH461131 | MH461133 |
| *Amorocoelophoma cassia* | MFLUCC 17–2283 | NG_066307 | NG_065775 | NR_163330 | MK434894 | MK360041 |  |
| *Neothyrostroma encephalarti* | CPC:35999 | MN567613 | N562105 |  |  | MN556831 |  |
| *Neothyrostroma encephalarti* | CBS:146037 |  |  |  |  | MN556830 |  |
| *Neomassaria formosana* | NTUCC 17-007 | MH714756 |  | MH714759 | MH714765 | MH714762 |  |
| *Neomassaria formosana* | NTUCC 17-013 | MH714757 |  | MH714760 | MH714766 | MH714763 |  |
| *Leucaenicola aseptata* | MFLUCC 17-2423 | NG_066309 | NR_163332 | NG_065776 | MK434891 |  |  |
| *Leucaenicola phraeana* | MFLUCC 18-0472 | NG_066317 |  | NG_065784 | MK434867 | MK360060 |  |
| *Leucaenicola osmanthi* | NTUCC 18-101-1 | MN908612 | MN908565 | MN908609 | MN915020 | MN918596 |  |
| *Leucaenicola osmanthi* | NTUCC 18-101-2 | MN908611 | MN908566 | MN908608 | MN915018 | MN918597 |  |
| *Leucaenicola osmanthi* | NTUCC 18-101-3 | MN908610 | MN908564 | MN908607 | MN915019 | MN918598 |  |
| *Paraphaeosphaeria arecacearum* | CBS 158.75 | JX496156 | JX496043 |  |  |  | JX496382 |
| *Paraphaeosphaeria minitans* | CBS 111750 | JX496130 | JX496017 |  |  |  | JX496356 |
| *Paraphaeosphaeria neglecta* | CBS 124078 | MH874872 | MH863348 |  |  |  | JX496378 |
| *Paraphaeosphaeria sporulosa* | strain CBS 391.86 | JX496195 | JX496082 |  |  |  | JX496421 |
| *Paraphaeosphaeria angularis* | CBS 167.70T | MH871317 | JX496047 |  |  |  | JX496386 |
| *Paraph. neglecta* | CBS 119637 | JX496138 | JX496025 |  |  |  | JX496364 |
| *Paraph. pilleata* | CBS 102207 | JX496126 | JX496013 |  |  |  | JX496352 |
| *Paraphaeosphaeria sardoa* | CBS 501.71 | MH872003 | MH860235 |  |  |  | JX496433 |
| *Paraphaeosphaeria sp.* | CBS 101464 | JX496125 | JX496012 |  |  |  | JX496351 |
| *Paraphaeosphaeria sporulosa* | CBS 105.76 | JX496127 | JX496014 |  |  |  | JX496353 |
| *Paraphaeosphaeria verruculosa* | CBS 263.85 | MH873567 | MH861879 |  |  |  | JX496398 |
| *Paraphaeosphaeria viridescens* | CBS 854.73 | MH872545 | JX496085 |  |  |  | JX496424 |
| *Paraphaeosphaeria rosae* | MFLUCC 17-2547 | MG829044 | MG828935 | MG829150 |  | MG829222 |  |
| *Paraphaeosphaeria pilleata* | SH ISS01F |  | KY048173 |  |  |  |  |
| *Paraphaeosphaeria parmeliae* | CBS 131728 | KP170722 | KP170654 |  |  | KP170679 | KP170703 |
| *Paraphaeosphaeria xanthorrhoeae* | CBS 142164 | KY979793 | KY979738 |  |  | KY979888 | KY979909 |
| *Paraphaeosphaeria rosicola* | MFLU 18-0108 | MG829047 | NR_157528 | MG829153 |  |  |  |
| *Paraphaeosphaeria viciae* | MFLU 15-1231 | KY397947 | NR_158840 | KY397948 |  |  |  |
| *Paraphaeosphaeria spartii* | MFLU:14 C0810 | KP711362 | KP711357 | KP711367 |  |  |  |
| *Paraphaeosphaeria sp.* | MFLUCC 15-0450 | KX954398 | KX965729 | KX986342 |  |  | KY197981 |
| *Karstenula rhodostoma* | CBS 690.94 | GU301821 |  | GU296154 |  |  |  |
| *Paraconiothyrium africanum* | STE-U 6316 |  | EU295650 | EU295654 |  |  |  |
| *Paraconiothyrium archidendri* | CBS 168.77 | JX496162 | JX496049 |  |  |  | JX496388 |
| *Paraconiothyrium cyclothyrioides* | CBS 972.95 | JX496232 | JX496119 | AY642524 |  |  | JX496458 |
| *Paraconiothyrium estuarinum* | CBS 109850 | JX496129 | JX496016 | AY642522 |  |  | JX496355 |
| *Paraconiothyrium archidendri* | C321 | MK347974 | MK347757 | MK347863 |  |  |  |
| *Paraconiothyrium fuckelii* | CBS 508.94 | JX496209 | JX496096 |  |  |  | JX496435 |
| *Paraconiothyrium fuckelii* | MFLUCC 130073 | KJ939281 | KJ939278 | KJ939284 |  |  |  |
| *Paraconiothyrium fungicola* | CBS 113269 | JX496133 | JX496020 | AY642527 |  |  | JX496359 |
| *Paraconiothyrium hawaiiense* | CBS 120025 | JX496140 | JX496027 | EU295655 |  |  | JX496366 |
| *Paraconiothyrium magnolia* | MFLUCC 100278 | KJ939283 | KJ939280 |  |  |  |  |
| *Austropleospora keteleeriae* | C453B | MK348021 | MK347802 | MK347910 |  |  |  |
| *Paraconiothyrium hawaiiense* | CPC 12268 |  | DQ885896 | EU295656 |  |  |  |
| *Paracamarosporium psoraleae* | CPC 21632 | KF777199 | KF777143 |  |  |  |  |
| *Pseudocamarosporium lonicerae* | MFLUCC 13-0532T | KJ813278 | KJ747047 | KJ819947 |  |  |  |
| *Pseudocamarosporium corni* | MFLUCC 13-0541T | KJ813279 | KJ747048 | KJ819946 |  |  |  |
| *Pseudocamarosporium cotinae* | MFLUCC 14-0624T | KP744505 | KP744460 | KP753964 |  |  |  |
| *Alloconiothyrium aptrootii* | 681F4R |  | MG751288 |  |  |  |  |
| *Xenocamarosporium acaciae* | CPC 24755 | KR476759 | KR476724 |  |  |  |  |
| *Xenocamarosporium acaciae* | C354 | MK347983 | MK347766 | MK347873 |  |  |  |
| *Neptunomyces aureus* | CMG10A |  | MK912119 |  |  | MK947998 | MK934130 |
| *Neptunomyces aureus* | CMG11 |  | MK912120 |  |  | MK947999 | MK934131 |
| *Cylindroaseptospora siamensis* | C329 | MK347976 | MK347760 | MK347866 |  |  |  |
| *Alloconiothyrium encephalarti* | CPC:35980 | MN567610 | MN562102 |  |  |  |  |
| *Letendraea cordylinicola* | MFLUCC 11-0148 | NG_059530 | NR_154118 |  |  |  |  |
| *Letendraea cordylinicola* | MFLUCC11_0150 | KM213999 | KM213996 | KM214002 |  |  |  |
| *Pithomyces chartarum* | CBS 329.86 | MH873651 | MH861960 |  |  |  |  |
| *Pithomyces chartarum* | CBS 805.72 | MH872302 | MH860611 |  |  |  |  |
| *Deniquelata barringtoniae* | MFLUCC 11-0422 | JX254655 | JX254654 | JX254656 |  |  |  |
| *Deniquelata barringtoniae* | MFLUCC 11-0257 | KM213997 | KM213997 | KM214003 |  |  |  |
| *Tremateia arundicola* | MFLU 16-1275 | KX274248 | KX274241 | KX274254 |  |  |  |
| *Paramassariosphaeria clematidicola* | MFLU 16-0172 | KU743207 | KU743206 | KU743208 |  |  |  |
| *Paramassariosphaeria anthostomoides* | CBS 615.86 | MH873693 | MH862005 | GU205246 |  |  |  |
| *Laburnicola centaureae* | MFLUCC 13-0601T | KU743192 | KX274239 | KU743193 |  | MT114405 |  |
| *Laburnicola dactylidis* | MFLUCC 16-0285T | KU743204 | KU743203 | KU743205 |  | MT114403 |  |
| *Laburnicola hawksworthii* | MFLUCC 13-0602T | KU743195 | KU743194 | KU743196 |  | MT114402 |  |
| *Spegazzinia tessarthra* | SH 287 | AB807584 | JQ673429 | AB797294 |  | MT114407 |  |
| *Neoshiraia taiwanensis* | NTUCC 18-091-5 | MT150600 | MT112280 | MT071207 | MT114405 | MT434762 | MT308610 |
| *Neoshiraia taiwanensis* | NTUCC 18-091-4 | MT071257 | MT112281 | MT071208 | MT114403 | MT434763 | MT308611 |
| *Neoshiraia taiwanensis* | NTUCC 18-091-1 | MT071258 | MT112282 | MT071209 | MT114402 | MT434764 | MT308612 |
| *Neoshiraia taiwanensis* | NTUCC 18-091-3 | MT071259 | MT112283 | MT071210 | MT114407 | MT743274 | MT308613 |
| *Neoshiraia taiwanensis* | NTUCC 18-091-2 | MT071260 | MT112284 | MT071211 | MT114406 | MT743275 | MT308614 |
| *Neoshiraia taiwanensis* | NTUCC 17-031 | MT071261 | MT112285 | MT071212 | MT114404 | MT743276 | MT308615 |
| *Neoshiraia camelliae* | NTUCC 18-092-1 | MT071262 | MT112286 | MT071213 | MT743267 | MT513982 | MT308616 |
| *Neoshiraia camelliae* | NTUCC 18-092-2 | MT071263 | MT112287 | MT071214 | MT743268 | MT513981 | MT308617 |
| *Pyrenochaetopsis americana* | NTUCC 17-033 | MT071264 | MT112288 | MT071215 | MT253564 | MT743277 | MT308618 |
| *Ectophoma pomi* | NTUCC 17-034 | MT071265 | MT112289 | MT071216 | MT267531 | MT587560 | MT308619 |
| *Didymella segeticola* | NTUCC 18-098 | MT071266 | MT112290 | MT071217 | MT301953 | MT561877 | MT308620 |
| *Paraphaeosphaeria camelliae* | NTUCC 18-095-1 | MT071267 | MT112291 | MT071218 | MT743269 | MT743278 | MT308621 |
| *Paraphaeosphaeria camelliae* | NTUCC 18-095-2 | MT071268 | MT112292 | MT071219 | MT743270 | MT743279 | MT308622 |
| *Paraconiothyrium camelliae* | NTUCC 18-096 | MT071269 | MT112293 | MT071220 | MT277330 | MT473955 | MT308623 |
| *Alloconiothyrium camelliae* | NTUCC 17-032-1 | MT071270 | MT112294 | MT071221 | MT232967 |  | MT308624 |
| *Alloconiothyrium camelliae* | NTUCC 17-032-2 | MT071271 | MT112295 | MT071222 | MT232965 |  | MT308625 |
| *Alloconiothyrium camelliae* | NTUCC 17-032-3 | MT071272 | MT112296 | MT071223 | MT232966 |  | MT308626 |
| *Leucaenicola camelliae* | NTUCC 18-093-1 | MT071273 | MT112297 | MT071224 | MT249023 | MT743280 | MT308627 |
| *Leucaenicola camelliae* | NTUCC 18-093-2 | MT071274 | MT112298 | MT071225 | MT249024 | MT743281 | MT308628 |
| *Leucaenicola taiwanensis* | NTUCC 18-094-2 | MT071275 | MT112299 | MT071226 | MT316124 | MT743282 | MT308629 |
| *Leucaenicola camelliae* | NTUCC 18-093-3 | MT071276 | MT112300 | MT071227 | MT346593 | MT743283 | MT308630 |
| *Leucaenicola taiwanensis* | NTUCC 18-094-1 | MT071277 | MT112301 | MT071228 | MT364370 | MT743284 | MT308631 |
| *Leucaenicola camelliae* | NTUCC 18-093-4 | MT071278 | MT112302 | MT071229 | MT374091 | MT743285 | MT308632 |
| *Amorocoelophoma camelliae* | NTUCC 18-097-1 | MT071279 | MT112303 | MT071230 | MT743271 | MT459143 |  |
| *Amorocoelophoma camelliae* | NTUCC 18-097-2 | MT071280 | MT112304 | MT071231 | MT743272 | MT459141 |  |
| *Amorocoelophoma camelliae* | NTUCC 18-097-3 | MT071281 | MT112305 | MT071232 | MT743273 | MT459142 |  |
| *Roussoella mexicana* | NTUCC 18-099-1 | MT071282 | MT112306 | MT071233 | MT211575 | MT497005 |  |
| *Roussoella mexicana* | NTUCC 18-099-2 | MT071283 | MT112307 | MT071234 | MT211576 | MT497006 |  |
| *Roussoella mexicana* | NTUCC 18-099-3 | MT071284 | MT112308 | MT071235 | MT416126 | MT497007 |  |
| *Neoshiraia taiwanensis* | NTUCC 18-091-6 | MT150602 | MT112309 | MT071236 | MT425440 | MT434766 | MT308633 |
| *Neoshiraia taiwanensis* | NTUCC 18-091-7 | MT150601 | MT112310 | MT071237 | MT425441 | MT434765 | MT308634 |
