# Supplementary material for: Species diversity of Pleosporalean taxa associated with Camellia sinensis (L.) Kuntze in Taiwan
Source: Sci Rep. 2020 Jul 29;10:12762. doi: 10.1038/s41598-020-69718-0 (PMC7391694; doi:10.1038/s41598-020-69718-0)
Supplement: Supplementary file 3 — Supplementary Information 3. [file 41598_2020_69718_MOESM3_ESM.docx]

**Species diversity of Pleosporalean taxa associated with *Camellia sinensis* (L.) Kuntze in Taiwan**

**Hiran A. Ariyawansa^1,*^, Ichen Tsai^1^, Kasun M. Thambugala^2^, Wei-Yu Chuang^1^, Shiou-Ruei Lin^3^, Wael N. Hozzein^4,5,^ Ratchadawan Cheewangkoon^6,7,*^**

^1^Department of Plant Pathology and Microbiology, National Taiwan University, College of Bio-Resources and Agriculture, Taipei City, 10617, Taiwan

^2^Department of Tea Agronomy, Tea Research and Extension Station, Taoyuan City, 32654, Taiwan

^3^Bioproducts Research Chair, Zoology Department, College of Science, King Saud University, Riyadh, 11451, Saudi Arabia

^4^Botany and Microbiology Department, Faculty of Science, Beni-Suef University, Beni-Suef, 62521, Egypt

^5^Genetics and Molecular Biology Unit, Faculty of Applied Sciences, University of Sri Jayewardenepura, Gangodawila, Nugegoda, Sri Lanka.

^6^Department of Entomology and Plant Pathology, Faculty of Agriculture, Chiang Mai University, Chiang Mai, 50200, Thailand

^7^Innovative Agriculture Research Centre, Faculty of Agriculture, Chiang Mai University, Chiang Mai, 50200, Thailand

*Corresponding authors: [ariyawansa44@ntu.edu.tw](mailto:ariyawansa44@ntu.edu.tw); ratchadawan.c@cmu.ac.th

Supplementary **TABLE 2.** Evaluation of alignment properties of genes and nucleotide substitution models used in the phylogenetic analyses of Pleosporales.

| Genes/loci | LSU | ITS | SSU | *rpb*2 | *tef*1 | *tub*2 |
| --- | --- | --- | --- | --- | --- | --- |
| Alignment strategy (MAFFT v6) | G-INS-1 | G-INS-1 | G-INS-1 | G-INS-1 +manual | G-INS-1 +manual | G-INS-1 +manual |
| Nucleotide substitution models for Bayesian analysis (determined by MrModeltest) | GTR+I+G | GTR+I+G | GTR+I+G | GTR+I+G | GTR+I+G | GTR+I+G |

Supplementary **TABLE 3.** Evaluation of alignment properties of genes and nucleotide substitution models used in the phylogenetic analyses of the Family Didymosphaeriaceae.

| Genes/loci | LSU | ITS | SSU | *tub*2 |
| --- | --- | --- | --- | --- |
| Alignment strategy (MAFFT v6) | G-INS-1 | G-INS-1 | G-INS-1 | G-INS-1 +manual |
| Nucleotide substitution models for Bayesian analysis (determined by MrModeltest) | GTR+I+G | SYM+I+G | GTR+I+G | K80+I+G |
